# Inhibitory effect of *Lonicera japonica flos* on *Streptococcus mutans* biofilm and mechanism exploration through metabolomic and transcriptomic analyses

**DOI:** 10.3389/fmicb.2024.1435503

**Published:** 2024-07-03

**Authors:** Lin Wang, Ping Liu, Yulun Wu, Hairun Pei, Xueli Cao

**Affiliations:** Beijing Technology and Business University, Beijing Advanced Innovation Centre for Food Nutrition and Human Health, Beijing, China

**Keywords:** *Lonicera japonica flos*, *S. mutans*, quorum sensing, metabolomic and transcriptomic, biofilm

## Abstract

**Introduction:**

*Streptococcus mutans* was the primary pathogenic organism responsible for dental caries. *Lonicera japonica flos* (LJF) is a traditional herb in Asia and Europe and consumed as a tea beverage for thousands of years.

**Methods:**

The inhibitory effect and mechanism of LJF on biofilm formation by *S. mutans* was investigated. The active extracts of LJF were validated for their inhibitory activity by examining changes in surface properties such as adherence, hydrophobicity, auto-aggregation abilities, and exopolysaccharides (EPS) production, including water-soluble glucan and water-insoluble glucan.

**Results and discussion:**

LJF primarily inhibited biofilm formation through the reduction of EPS production, resulting in alterations in cell surface characteristics and growth retardation in biofilm formation cycles. Integrated transcriptomic and untargeted metabolomics analyses revealed that EPS production was modulated through two-component systems (TCS), quorum sensing (QS), and phosphotransferase system (PTS) pathways under LJF stress conditions. The sensing histidine kinase VicK was identified as an important target protein, as LJF caused its dysregulated expression and blocked the sensing of autoinducer II (AI-2). This led to the inhibition of response regulator transcriptional factors, down-regulated glycosyltransferase (Gtf) activity, and decreased production of water-insoluble glucans (WIG) and water-soluble glucans (WSG). This is the first exploration of the inhibitory effect and mechanism of LJF on *S. mutans*, providing a theoretical basis for the application of LJF in functional food, oral health care, and related areas.

## 1 Introduction

With the acceleration of contemporary lifestyles and the proliferation of highly processed foods, individuals increasingly sought sugar to enhance their gustatory experience. Dental caries, a biofilm-mediated oral disease, were closely associated with suboptimal dietary habits and the consumption of high-sugar foods (Chen et al., 2021; Park et al., 2023). As a result, dental caries remained a common oral health issue over time. *Streptococcus mutans* (*S. mutans*) was the primary pathogenic organism responsible for dental caries, as biofilm formation was the main virulence factor (Barran-Berdon et al., 2020; Bhatt et al., 2020). Therefore, identifying and understanding the main virulence factors of *S. mutans* that form biofilm becomes crucial for preventing dental caries.

Biofilm represents a structured bacterial community enveloped by an extracellular polymeric matrix (Matsumoto-Nakano, 2018). The accepted theory of plaque formation was roughly divided into three stages. Firstly, glycoproteins in saliva adhered to the tooth surface to form an acquired membrane. Next, *S. mutans* cells adhered to this membrane. Once firmly attached, further adhesion between bacteria led to aggregation and the formation of a biofilm, commonly known as dental plaque (Chen et al., 2019; Li J. et al., 2020). Once formed, the biofilm could resist environmental stress. Exopolysaccharides (EPS) were the main components of the biofilm, and along with extracellular proteins and water, they formed extracellular polymers. The extracellular matrix within the biofilm provided protection from harmful factors. *S. mutans* was the leading producer of EPS and could encode multiple exoenzymes, such as glycosyltransferases (Gtf) and glucan-binding proteins. Gtfs were produced inside the cells and secreted outside, meaning they were present in the extracellular medium. Sucrose was the substrate of Gtfs and was used to synthesize glucans (Sutherland, 2001; Hoshino and Fujiwara, 2022). Glucans were the main EPS in biofilm and provided additional microbial binding sites (Schilling and Bowen, 1992; Steinberg et al., 1993; Venkitaraman et al., 1995). Despite the emergence of new strategies such as secondary messenger signaling pathway, quorum sensing pathway, and eDNA, controlling EPS production is still the main way to inhibit biofilm formation.

For treatment of cariogenic biofilms, CH was considered the gold standard in dentistry and was recommended to prevent dental caries (Persson et al., 2007). However, conventional medications, including CH, cipyridine, triclosan, etc., exhibited limited efficacy over a short duration. Long-term use of these therapies could decrease oral microbial diversity (Scully and Greenman, 2012; Pragman et al., 2021). Thus, they are not suitable for daily prevention. There is an urgent need for treatments of oral diseases with low side effects. The utilization of phytochemicals presented a promising approach, as evidence indicated the impact of plant extracts on the virulence of *S. mutans*. For instance, *Kaffir Lime leaf* exhibited an anti-biofilm effect by regulating genes associated with biofilm formation (Rather et al., 2021), *Emblica officinalis* extract reduced surface properties and glucan synthesis, and *Achyranthes aspera* extract inhibited Gtf activity (Murugan et al., 2013). Overall, phytomedicine are considered a valuable source of inhibitory substances involved in biofilm formation.

*Lonicerae japonica flos* (LJF, known as Jinyinhua in Chinese), the dried flowers or flower buds of *Lonicera japonica Thunberg*, is a medicinal-edible herb antibacterial, antiviral, and anti-inflammatory properties that have been well-studied and widely used for thousands of years (Li et al., 2014; Cai et al., 2020; Zheng et al., 2022). It is also consumed as a tea beverage in Asia and Europe because of its unique aroma and flavor (Li et al., 2014; Yang et al., 2017). Moreover, chlorogenic acid, the main component of LJF, has been shown in previous studies to inhibit the biofilm formation of *Pseudomonas*, *Staphylococcus aureus*, and *Salmonella enteritidis* (Sun et al., 2021, 2022; Wang L. et al., 2023). This suggests that LJF may have inhibitory effects on the biofilm formation of *S. mutans*. However, the current assessment of anti-biofilm effect is insufficient and the inhibition mechanism lacks comprehensiveness.

In the last years, integrated transcriptomic and metabolomic analyses provide a molecular-scale perspective on the response of crops to pesticides (Liu and Zhu, 2020), the effects on the interaction of tea plant and *Colletotrichum camelliae* (Lu et al., 2020). Integrated multi omics analyses aroused a high interest because of their potential for the identification of molecular features and target discovery. In the present study, the inhibitory effect of LJF samples from different origins on *S. mutans* was examined. The active extracts were evaluated for the inhibitory activity in terms of changes in surface properties, EPS production, and quorum-sensing (QS) signaling molecule AI-2. Integrated metabolomics and transcriptomic analyses were applied to investigate the differentiation of metabolites and gene expression in biofilms, elucidating inhibitory mechanisms of LJF on biofilm formation.

## 2 Materials and methods

### 2.1 Materials and reagents

**Figure 1:**
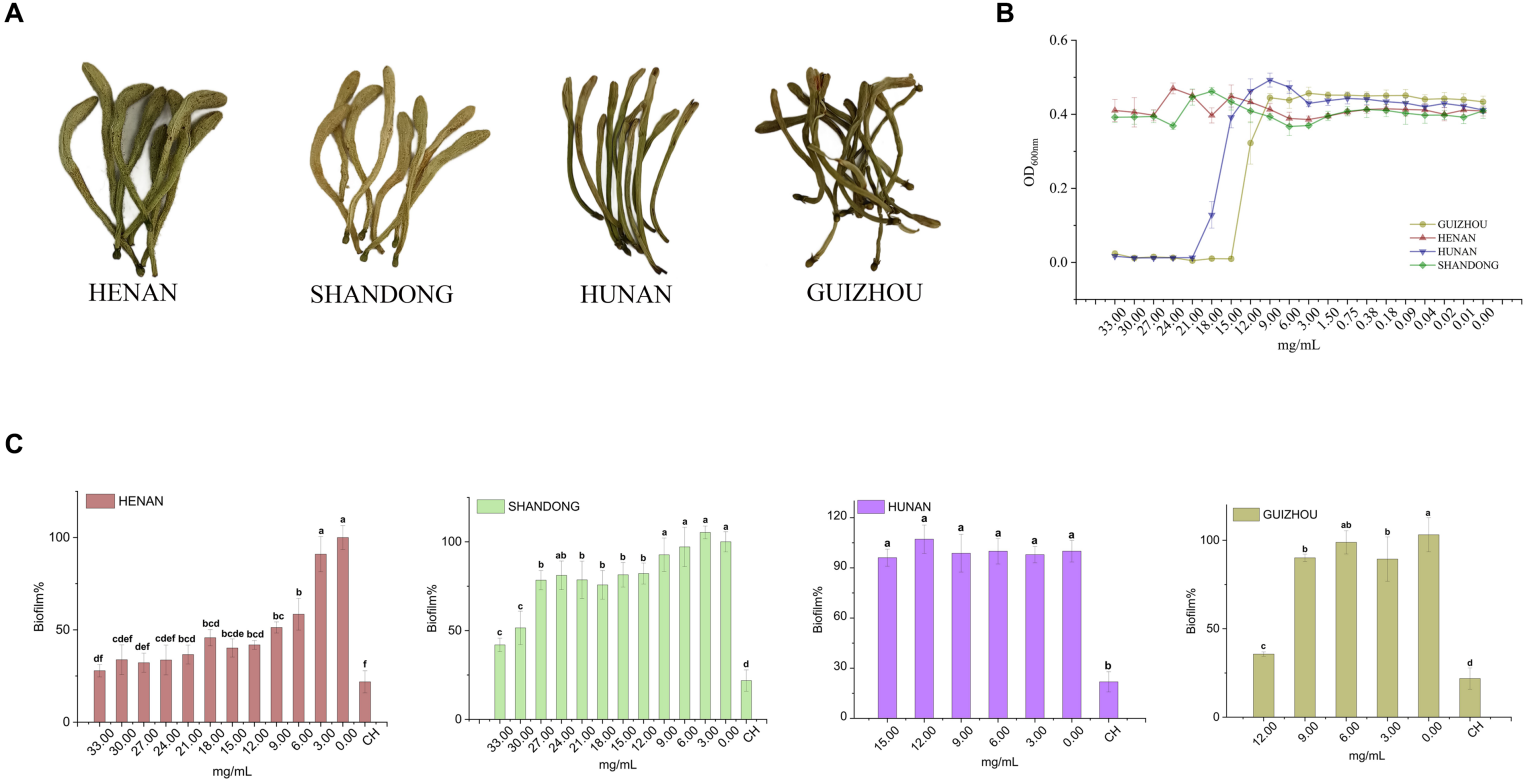
Inhibitory effect of different cultivars of LJF on planktonic *S. mutans* and biofilm production. **(A)** Four cultivars of LJF. **(B)** Inhibitory effect of different LJF cultivars on *S. mutans.* **(C)** Inhibitory effect of different LJF cultivars on *S. mutans* biofilm production. For the letters a–f, mean values (*n* = 5) with different letters within the same column are significantly different (*p* < 0.05).

Dried LJF samples were collected from four different origins in 2021. Sample information is provided in Supplementary Table S1, and plant morphology is shown in Figure 1A. *S. mutans* strain UA159 (ATCC700610) was purchased from the China General Microbiological Culture Collection Center and sub-cultured under anaerobic conditions for experiments. The anaerobic environment was maintained in a 2.5 L anaerobic incubator (D-110, Mitsubishi, Japan) using a disposable AnaeroPack (MGC AnaeroPack Series D-04, Mitsubishi, Japan). For biofilm formation, 1% sucrose was added to Brain Heart Infusion medium (BHIS).

### 2.2 Preparation of LJF extract

LJF extracts were prepared as previously elucidated (Wang S. et al., 2023). Each sample (10.0 g) was extracted with 100 mL of 60% aqueous methanol under ultrasonication, yielding approximately 1 g of extract from 10 g of dry sample.

### 2.3 Minimum inhibitory concentration assay of LJF and biofilm formation of *Streptococcus mutans*

The minimum inhibitory concentration (MIC) of the LJF extract against *S. mutans* was tested as described with some modification (Li et al., 2018). Firstly, 1.5 g of LJF extract was dissolved in 45 mL sterile BHIS, and the bacteria-free solution was obtained by filtering through a 0.22-μm membrane. Then, 190 μL BHIS containing diluted LJF extract (30.00, 27.00, 24.00, 21.00, 18.00, 15.00, 12.00, 9.00, 6.00, 3.00, and 0.00 mg/mL) was added to a 96-well plate. Each well was supplemented with 10 μL *S. mutans* culture (OD_600nm_ 0.17). Five replicates were performed at each concentration. Positive control groups contained BHIS culture, while background control groups were supplemented with 10 μL saline instead of *S. mutans* culture. After 16 h of incubation, data was read at 600 nm using a microplate reader (Infinite M200PRO, TESCAN, Mannedorf, Switzerland). MIC was determined as the lowest concentration with no inhibitory effect. Biofilm formation was assessed as described (De Jesus and Dedeles, 2020), with data read at 590 nm using the microplate reader, and the percentage of biofilm formation calculated.

### 2.4 Morphology of *Streptococcus mutans* biofilm

First, 1.9 mL of 21.00 mg/mL LJF was added to each well containing a slice (φ15 mm, NEST, Wuxi, China) in a 24-well plate. Then, 100 μL of a bacterial suspension of *S. mutans* (OD_600nm_ 0.17) was added to each well. The plate was incubated at 37°C for 16 h. After incubation, the biofilm was fixed as described (Jiang et al., 2022). Images were obtained using SEM (SU8020, Hitachi, Japan). For Fourier Transform Infrared Spectroscopy (FTIR) (Nicolet IS10, Thermo Fisher, USA), 1 mg of biofilm from each slice was mixed with 100 mg of KBr and ground (Chang et al., 2021; Howard et al., 2023).

### 2.5 Analysis of the main components of *Streptococcus mutans* biofilm

#### 2.5.1 Viability of biofilm-entrapped cells

After incubation in the 96-well plate, the supernatants were removed, and the biofilm formed on the plates was rinsed twice with PBS. Then, 200 μL of 0.5 mg/mL 3,-3′-bis (4-methoxy-6-nitro) benzene sulfonic acid (XTT) was added to each well and incubated in an opaque black plastic bag at 37°C for 4 h. The absorbance at 490 nm was recorded as viability, as described (Kim et al., 2022).

#### 2.5.2 Contents of EPS

The water-soluble glucan (WSG) and water-insoluble glucan (WIG) of the *S. mutans* biofilms were extracted as previously described with some modifications (Zhang H. et al., 2022; Rudin et al., 2023). The detailed steps are shown in Supplementary Figure S1. The absorbance of WSG and WIG was measured using the anthrone method.

### 2.6 Surface properties of *Streptococcus mutans* in biofilm

The adherence of cells in each group was measured using the method of Islam et al. (2008). The hydrophobicity of biofilm cells in each group was determined according to the method of Ullah et al. (2023). The aggregation capacity (AC) was evaluated as described by Kolenbrander (Bosch et al., 2012). Detailed steps for these determinations are shown in Supplementary Figure S1. Data was read at 600 nm using a microplate reader.

### 2.7 Autoinducer-2 bioassay

Autoinducer-2 (AI-2) was determined using methods previously described, with some modifications. To begin the assay, 9.0 mL of a *V. harveyi* BB170 cell culture diluted 1:100 was placed into a set of sterile plastic rocker tubes. Then, 1.0 mL of tested samples, negative control, positive control, and chemically synthesized pure AI-2 (10 μM) were added separately to each tube (Supplementary Figure S2). The luminescence value (RL) (SpectraMax^®^i3, Molecular Devices, Shanghai, China) was measured in photons/s mode every 60–120 min for a total of 8 h. AI-2 content was determined as described (Vilchez et al., 2007; Cuadra et al., 2016; Muras et al., 2018; Zhang Y. et al., 2022). The relative luminescence intensity (RLI) and the content of AI-2 was determined as follows:


$$RLI=\frac{RL_{\mathrm{sample}}-RL_{\mathrm{medium}\;\mathrm{control}}}{RL_{BB170}-RL_{\mathrm{AB}\;\mathrm{medium}}}$$

$$\mathrm{AI}-2=\frac{RLI_{\mathrm{sample}}\times 10_{10\;\mu M\;\mathrm{AI}-2}}{RLI_{10\;\mu M\;\mathrm{AI}-2}}$$

### 2.8 Metabolomics analysis of *Streptococcus mutans* biofilm

First, 10 mL of 21.00 mg/mL LJF was added into a tissue culture-treated dish (NEST, Cat.No:704001, Jiangsu, China). Then, 1,000 μL bacterial suspension was added into each dish. After 16 h incubation, dishes were rinsed with sterile water twice. Biofilms cultured in the same volume of non-LJF BHIS were set as control group. An amount of 50 mg of each biofilm sample was weighed accurately and added into 2 mL centrifuge tubes. Metabolites extraction and MS data collection were according to the previous method (Li et al., 2024). Metabolomics data have been deposited to the EMBL-EBI MetaboLights database: with the identifier MTBLS9711. The complete dataset could be accessed at “https://www.ebi.ac.uk/metabolights/MTBLS9711”.

### 2.9 Transcriptomic analysis of *Streptococcus mutans* biofilm

A total of 100 mg biofilm cells were collected using 1 mL of PBS from dishes. Total RNA was isolated as described (Li et al., 2019). Whole mRNAseq libraries were generated by Guangdong Magigene Biotechnology Co., Ltd. Result of the quality control can be seen from Supplementary Table S3. Differentially expressed genes (DEG) were selected according to the criterion: genes with FDR ≤ 0.05 and |log2(fold change)| ≥ 1. Enrichment analysis of differentially expressed genes were performed by Heml, referred to the website of KEGG. Pathways with FDR ≤ 0.05 were considered significantly enriched. The RNA-seq datasets are available at the NCBI Sequence Read Archive (SRA) database under accession number PRJNA1119033. The complete dataset could be accessed at “https://www.ncbi.nlm.nih.gov/sra/PRJNA1119033”.

### 2.10 Quantitative reverse transcriptional polymerase chain reaction (qRT-PCR)

Primers for target genes were designed for biofilm formation-related DEGs in *S. mutans*. Primers were synthesized by Ruiboxingke Biotechnology Co. Ltd. (Beijing, China) and were shown in Supplementary Table S2. The PCR reactions were conducted according to the previous described method (Park et al., 2023).

### 2.11 Statistical analysis

A one-way analysis of variance (ANOVA) was conducted using SPSS to determine significant differences in biofilm formation properties, with a significance level set at *p* < 0.05. Data were visualized using Origin 9. Chemometrics for metabolomics and transcriptomics were performed in R (4.3.1) and Biodeep.^1^ Finally, a mechanism diagram was created using Adobe Illustrator.

## 3 Results

### 3.1 Effects of different cultivars on *Streptococcus mutans* biofilm formation

The inhibitory effect of LJF extracts from different cultivars was initially evaluated through a series of concentration tests to determine their impact on the planktonic growth of *S. mutans*. As shown in Figure 1B, LJF samples from Hunan and Guizhou exhibited no inhibitory effect at concentrations below 15.00 mg/mL and 12.00 mg/mL, respectively, indicating MIC values higher than these concentrations. Similarly, LJF samples from Henan and Shandong showed no inhibitory effect at concentrations lower than 33.00 mg/mL, suggesting an MIC higher than 33.00 mg/mL. The antibacterial efficacy of Guizhou and Hunan extracts were significantly superior to that of the other two extracts.

However, as depicted in Figure 1C, the crystal violet staining assay revealed that Henan cultivar exhibited a robust dose-dependent inhibition of biofilm formation at concentrations below MIC, demonstrating an impressive inhibition rate of 72% (compared to control group) at 33.00 mg/mL. This rate was slightly below the positive control effect achieved by CH at 12.5 μM (79%). The Shandong cultivar displayed an inhibition rate of approximately 50% within the range of 30.00–33.00 mg/mL. In contrast, the Guizhou cultivar showed a modest inhibitory effect, while the Hunan cultivar exhibited no discernible inhibitory effect on biofilm formation at concentrations below the MIC, despite its demonstrated inhibitory effect on planktonic *S. mutans* growth. Consequently, the Henan cultivar was selected for subsequent investigations due to its superior performance in biofilm inhibition compared to other tested varieties.

### 3.2 Effect on the biofilm characteristics of *Streptococcus mutans*

#### 3.2.1 Morphology analysis

**Figure 2:**
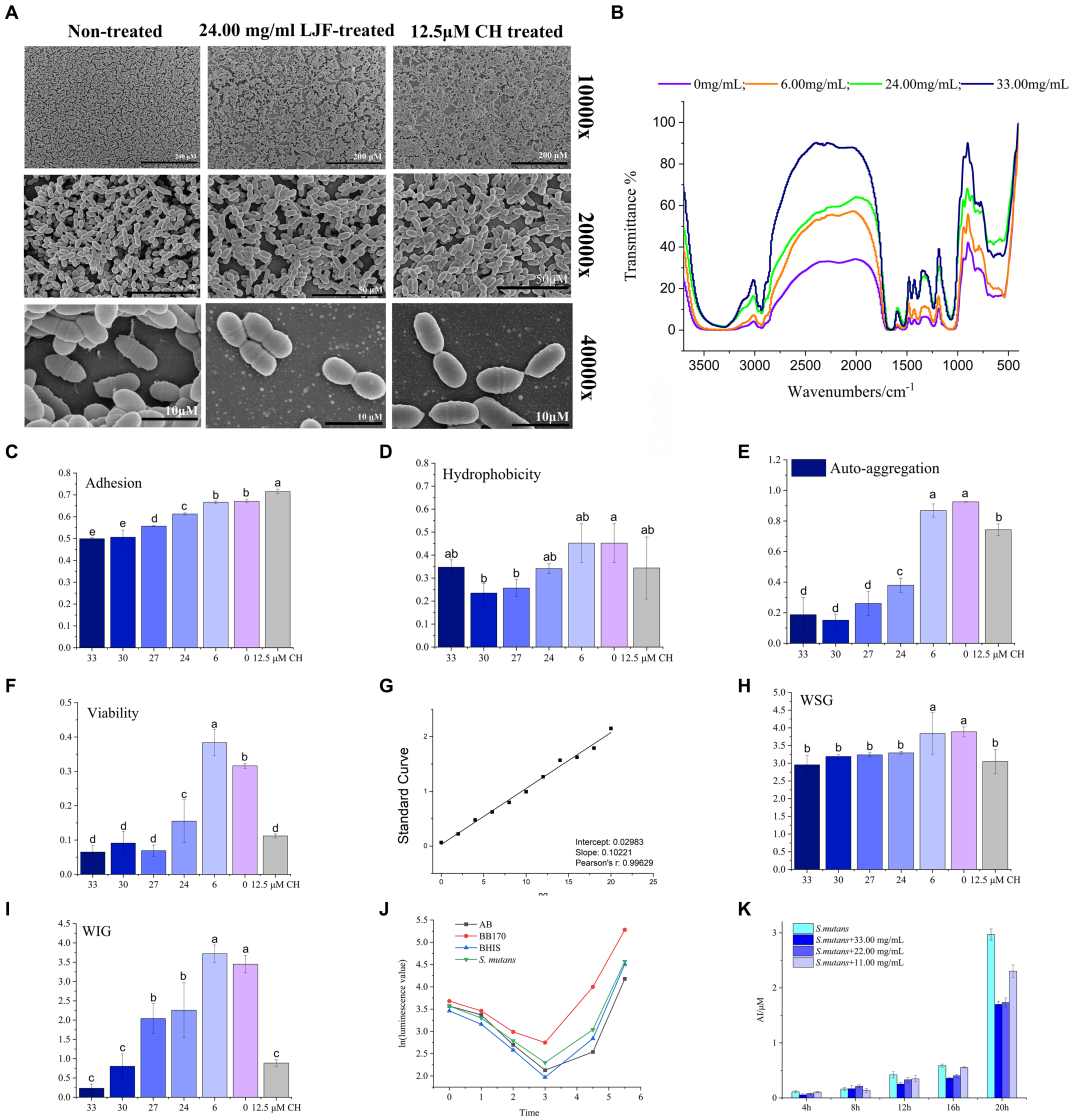
Effect of LJF on *S. mutans* biofilm. **(A)** Microstructure of *S. mutans* biofilm. **(B)** FTIR of *S. mutans* biofilm (Blank line: non-LJF treated sample; Yellow line: 6.00 mg/mL LJF inhibition sample; Light blue line: 21 mg/mL LJF inhibition sample; Pink line: 33.00 mg/mL LJF inhibition sample). **(C)** Effect of the LJF on the adhesion of *S. mutans* (*p*<0.05). **(D)** Effect of the LJF on the hydrophobicity of *S. mutans* (*p* < 0.05). **(E)** Effect of the LJF on the self-aggregation of *S. mutans* (*p* < 0.05). **(F)** Viability of bacteria in *S. mutans* biofilm (*p* < 0.05). **(G)** Standard curve of glucan. **(H)** Water soluble glucans (WSG) production of *S. mutans* biofilm (*p* < 0.05). **(I)** Water insoluble glucans (WIG) production of *S. mutans* biofilm. **(J)** AI-2 production of *S. mutans*. **(K)** Inhibitory effect of LJF on AI-2 production of *S. mutans*. Colors of column indicated the different concentration of LJF. For the letters a–e, mean values (*n* = 4) with different letters within the same column are significantly different (*p* < 0.05).

In control group, clusters of *S. mutans* exhibited a uniformly distributed multilayer and reticular biofilm (Figure 2A). But when cultured in the concentration of LJF and CH (24.00 mg/mL and 12.5 μM, respectively), the density of biofilm decreased, and the network structure was damaged to varying degrees. Biofilms in treated group displayed wider channels compared to biofilm in control group, such as fewer bacteria and channels, primarily attributed to the underdeveloped biofilm structure. Additionally, bacteria were arranged in a three-dimensional stacking pattern in both control group and treated group. It means that cell membrane permeability remained unaltered in LJF group at this concentration.

FTIR spectra of biofilm in control group and LJF group was measured to determine changes biofilm structure, chemical groups and compounds of unknown substances of biofilms, as presented in Figure 2B. Compared to the black line, the other three lines were narrow at 3,600–3,000 cm^−1^, 3,000–2,800 cm^−1^, 1,600–1,500 cm^−1^, and 1,200–1,000 cm^−1^. There is a broad peak at 3,600–3,000 cm^−1^. Maybe it caused by the hydration of EPS in biofilm and surface polysaccharide of biofilm cells. Additionally, a peak at 3,000–2,800 cm^−1^ corresponded to the C-H of EPS. A peak at 1,600–1,500 cm^−1^ was assigned to the C=O of polysaccharides. Peaks at 1,200–1,000 cm^−1^ were maybe caused by two kinds of C-O stretching vibration. The morphological analysis using SEM and FTIR indicated structural alterations in the biofilm structure of LJF-treated groups compared to the control group.

#### 3.2.2 Surface properties of biofilm cells

The main surface properties of biofilm include adherence, hydrophobicity, and auto-aggregation ability. LJF effectively inhibited the adherence of *S. mutans* biofilm in a dose-dependent manner (Figure 2C). Adherence of cells is likely attributed to the hydrophobic interaction between cells and the contact surface. Both LJF-treated and CH-treated groups exhibited lower hydrophobicity compared to the control group (Figure 2D) (*p* < 0.05). Auto-aggregation refers to the phenomenon of cellular aggregation in a bacterial culture, indicating the interaction of the mass and the rapid propagation of bacteria during the culture process. There was less auto-aggregation in the LJF-treated and CH-treated groups than in the control group (Figure 2E) (*p* < 0.05). Inhibition at 24.00–33.00 mg/mL occurred in a dose-dependent manner. Cell clumping properties were mainly regulated by surface EPS.

### 3.3 Effect on the main components of *Streptococcus mutans* biofilm

#### 3.3.1 Viability of biofilm-entrapped cells

The migratory capacity of the biofilm is contingent upon the bacterial activity within it. The XTT method represents a widely employed approach for assessing bacterial metabolic function. The viability of biofilm-entrapped cells significantly decreased in the presence of LJF, comparable to that of CH treatment (Figure 2F). This suggested that the metabolic capability of *S. mutans* biofilm decreased under LJF treatment.

#### 3.3.2 Extracellular polysaccharides of biofilms

As the survival of living bacteria in biofilm primarily relies on the polysaccharide matrix and dead bacteria for protective measures, glucan polymers play a crucial role in maintaining the three-dimensional structure of biofilms. EPS was the main component of polysaccharide matrix and primarily consist of two types of glucans, WSG and WIG. Figure 2G illustrated the standard curve used for quantitative detection of polysaccharides. As depicted in Figures 2H,I, both WSG and WIG production exhibited significant inhibition compared to the control group (*p* < 0.05). The application of LJF resulted in a significant reduction of 50–90% in WIG production, which is particularly noteworthy.

### 3.4 Effect on the AI-2 production

The LuxS/AI-2 quorum sensing (QS) system has been widely reported to play a role in biofilm formation in *S. mutans*. It can been seen from Figure 2J, there was low expression of AI-2 within the first 4 h, followed by a gradual increase. Compared to the control group (BHIS without *S. mutans*), the secretion of AI-2 by *S. mutans* was significantly higher in the group of inoculated BHIS. The observed trend exhibited a similar pattern to that of the positive control, indicating successful production of the AI-2 signal molecule by *S. mutans*. To investigate the impact of LJF on the LuxS/AI-2 QS system, we assessed changes in AI-2 secretion by *S. mutans* at different concentrations of LJF. Notably, presence of LJF resulted in decreased production of AI-2 by *S. mutans* (Figure 2K), confirming its inhibitory effect on biofilm through LuxS/AI-2.

In all, LJF-induced changes in surface features and EPS formation of *S. mutans* biofilm were confirmed. LuxS/AI-2 was identified as one of the inhibitory pathways associated with this phenomenon. In fact, the unique composition and characteristics of biofilms make combating biofilm infections extremely difficult due to their multi-microbial nature, which results in synergistic tolerance of multiple resistance mechanisms. Single-target treatments are usually ineffective against biofilm formation (Seneviratne et al., 2020). Therefore, we hypothesized that the inhibition of biofilm formation by LJF exhibited a multi-target mechanism.

### 3.5 Metabolomics analysis

**Figure 3:**
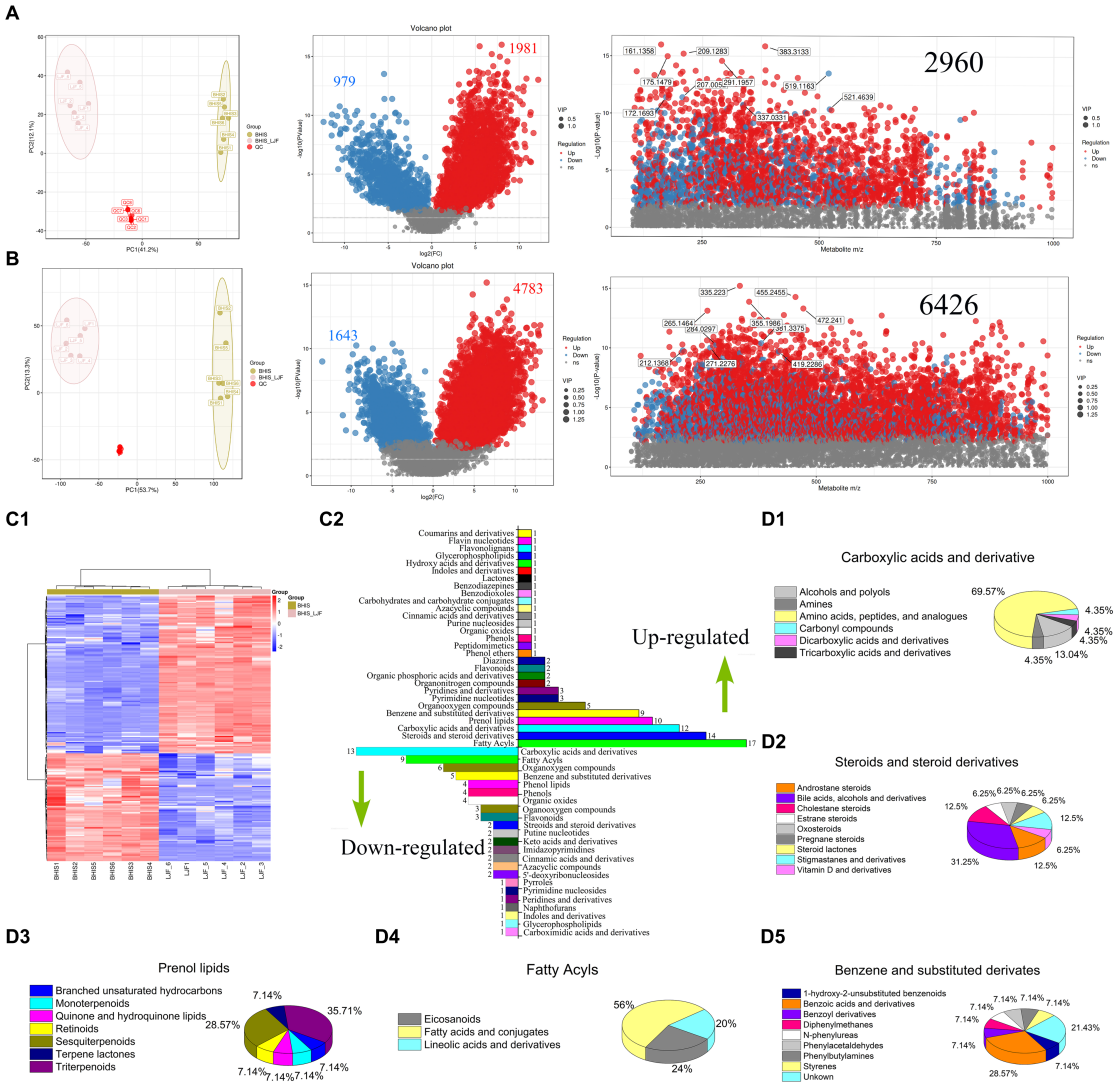
Differential analysis and metabolite identification. **(A)** Differential analysis in ESI positive mode. **(B)** Differential analysis in ESI negative mode. In the volcano plots, each compound is represented in red (up-regulated) or blue (down-regulated). In the scatter plot, the horizontal coordinate indicates the m/z of the compound. The ordinate represents the logarithm of the *p*-value. Each dot represents a compound. The red dot represents up-regulate and the blue dot represents down-regulate. The grey dot represents the metabolite that is below the screening criteria. The dot size represents the VIP value. **(C)** Cluster analysis by the metabolites identified in MS/MS mode. Upregulated metabolites were the right column. Down-regulated metabolites were left columns. **(D)** Composition of the main metabolite classes.

Untargeted metabolomics analysis was performed to achieve profiling changes of all metabolites with the exclusion of antibacterial function. The variation of critical metabolites revealed the regulated metabolic pathway. The UPLC-Qtof MS system was employed to harvest a total of 6,803 and 11,695 metabolites in the positive and negative modes, respectively. Principal component analysis (PCA) of the positive mode metabolome data (Figure 3A) revealed that PC-1 accounted for 41.2% of the variance, while PC-2 explained 12.1%, primarily contributing to the observed differences along PC-1 axis. In the negative mode (Figure 3B), PC-1 accounted for 53.7% of the variance, with PC-2 explaining 13.3%. The volcano plot depicted variations among all metabolites, where differential expressed metabolites (DEMs) were selected based on *p* < 0.05 and VIP ≥ 1.0 criteria: up-regulated metabolites were represented by red dots, down-regulated ones by blue dots, and grey dots indicated those not meeting screening criteria. In positive mode, a total of 1981 compounds were upregulated while 979 compounds were down-regulated; in negative mode, there were 4,783 up-regulated compounds and 1,643 down-regulated compounds identified separately as DEMs.

Therefore, we further identified key metabolites by integrating data from open-source databases and local databases, and explored the metabolic pathways associated with biofilm formation capacity. In total, 394 differentially expressed metabolites (DEMs) were identified using secondary mass spectrometry, and their clustering analysis is shown in the heatmap (Figure 3C1). The information of these identified metabolites can be found in Supplementary Table S4. Figure 3C2 illustrates the up-regulated and down-regulated DEMs, which include fatty acyls, carboxylic acids and derivatives, steroids and steroid derivatives, benzene and substituted derivatives, prenol lipids, organooxygen compounds, as well as pyrimidine nucleotides. The subclasses of these compounds are presented in Figures 3D1–D5. Most of DEMs were the metabolites regulated in alanine, aspartate and glutamate metabolism, biosynthesis of various secondary metabolites, lysine degradation, glyoxylate and dicarboxylate metabolism, two-component system (TCS), pyrimidine metabolism, purine metabolism, and ABC transporters. At the metabolic level, all but ABC transporters were the pathways that inhibited biofilm formation and significantly regulated by DEMs.

### 3.6 Transcriptomic analysis

**Figure 4:**
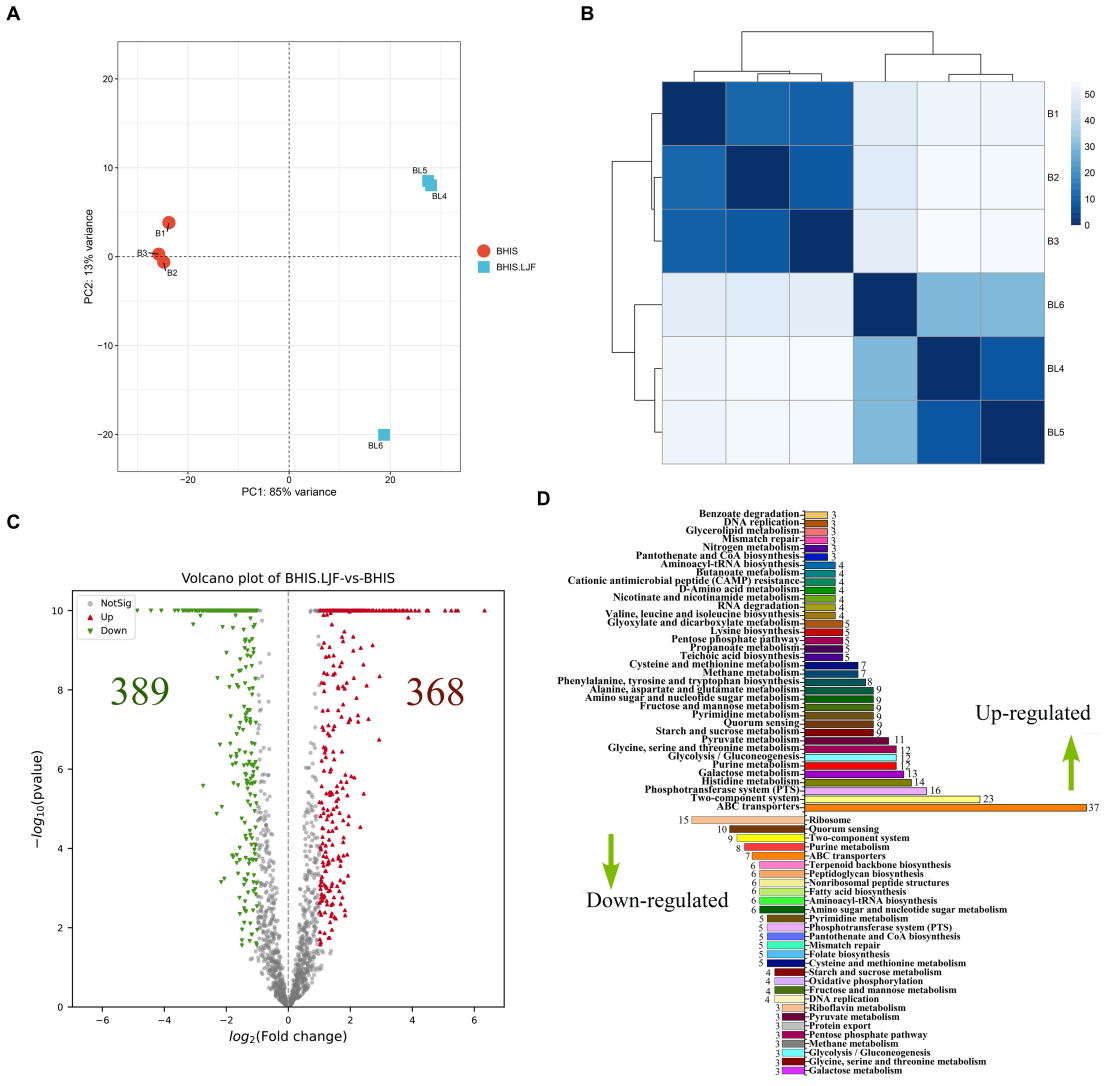
Transcriptomic analysis. **(A)** PCA score plot. **(B)** Distance heatmap. **(C)** Volcano plots of DEG on RNA-seq analysis of untreated and LJF-treated *S. mutans*. Each gene is represented in red (upregulated) and green (downregulated) and they are DEGs with >2 fold change and *p* < 0.05. **(D)** Numbers of DEGs and RT-PCR validation. Up-regulated DEGs were the right columns and down-regulate DEMs were the left columns.

The LJF-treated and control groups exhibited a conspicuous disparity, characterized by a substantial variance in PC-1 and a considerable dissimilarity over long distances (Figures 4A,B). DEGs with |Fold change| (|FC|) ≥2, *p*-value <0.05, FDR ≤ 0.05 were selected. The LJF group exhibited a total of 389 up-regulated genes, while the remaining 368 genes were down-regulated (Figure 4C). The prominently up-regulated genes were associated with ABC transporters, TCS, and phosphotransferase system (PTS). Conversely, the down-regulated genes primarily participated in ribosome function, QS, and TCS (Figure 4D). We further validated the altered expression of these genes induced by LJF using quantitative real-time PCR assay. This finding is consistent with the mRNA-seq analysis results for gene expression levels (Supplementary Table S5). These screened genes for RT-PCR validation involved in biofilm formation. The expression levels of the aforementioned genes were consistent with the results obtained from mRNA-seq analysis (Supplementary Figure S3), thereby establishing confidence in the enrichment analysis. Most of DEGs were enriched in the pathways of TCS, purine metabolism, PTS, histidine metabolism, glycine, serine and threonine metabolism, galactose metabolism, fructose and mannose metabolism. At the level of transcription, PTS, histidine metabolism, galactose metabolism, glycine, serine and threonine metabolism were the most regulated pathways by LJF.

Mechanism analysis based on transcriptomics and metabolomics obtained several pathways that regulated biofilm formation, but the information obtained by the two kinds of omics analysis could not be completely consistent, and the target information could not be judged. Therefore, it is necessary to integrate transcriptomics and metabolomics analyses to focus on the target of LJF inhibiting biofilm formation.

### 3.7 Conjoint analysis of transcriptomic and metabolomics data

**Figure 5:**
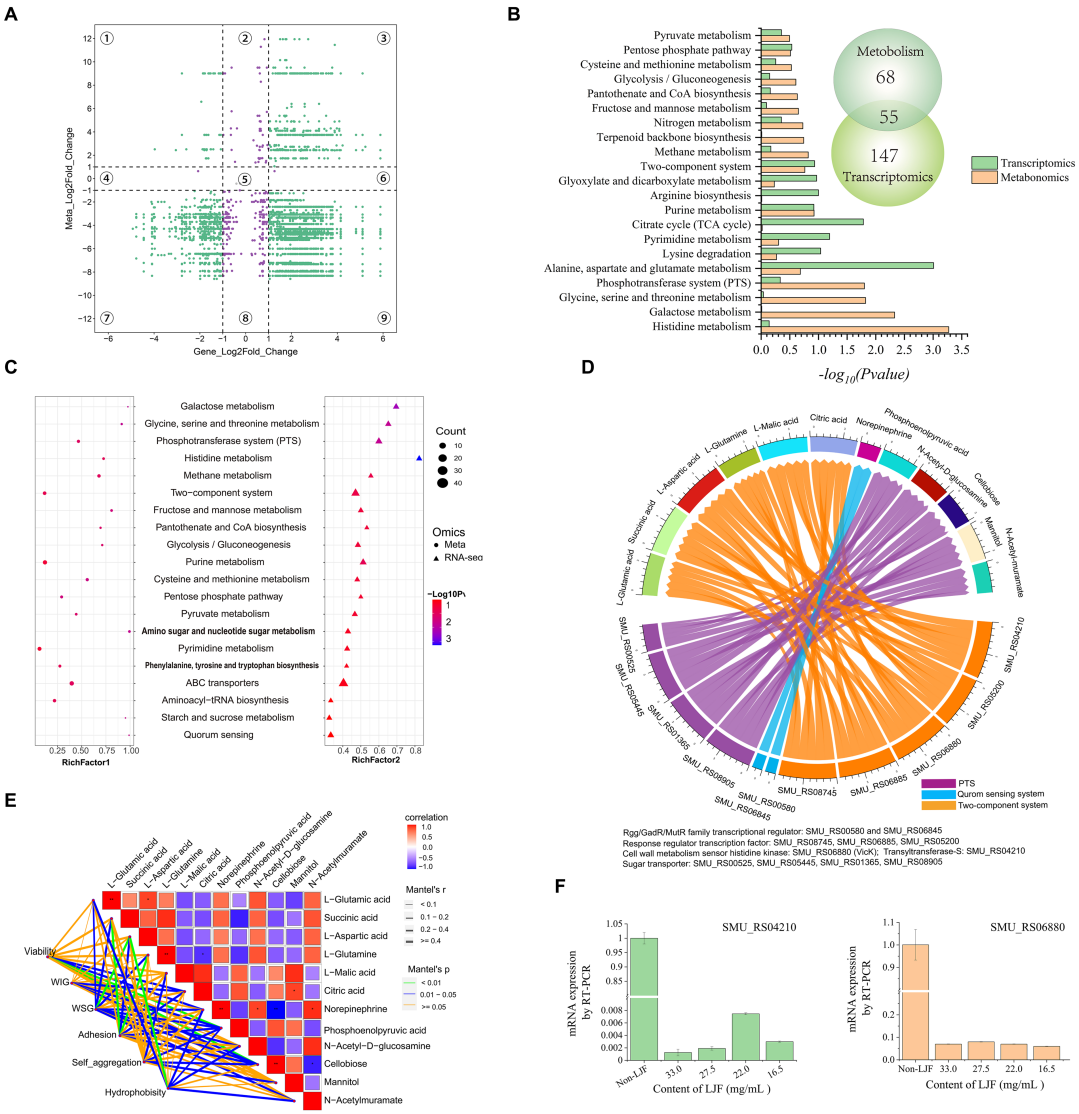
The conjoint analysis of metabolomics and transcriptomics. **(A)** A four-image map to analyze the relationship between DEMs and DEGs. **(B)** KEGG pathway analysis of DEGs and DEMs. **(C)** KEGG enrichment scatter plot. **(D)** Chord diagram of metabolites and genes. **(E)** Association analysis of biofilm character and biofilm character. **(F)** Gene expression level of SMU_RS04210 (*gtfS*) and SMU_RS06880 (*vicK*).

A four-image map of DEGs and DEMs, visually represented the correlation between gene expression and metabolite changes (Figure 5A). Purple points in parts two, four, five, six, and eight indicated non-correlated DEMs and DEGs, while green points represented significant co-differentials with a screening criterion of |FC| ≥ 2. There were more points in the parts that indicated correlated DEMs and DEGs. These findings established a foundation for further exploration of the anti-biofilm mechanism of LJF. KEGG pathway enrichment analysis identified 55 pathways in both transcriptomic and metabolomic analyses (Figure 5B). The transcriptomic results supported the metabolomic analysis. Pathway enrichments of DEMs and DEGs are presented in Figure 5C based on *p*-value, impact (metabolomic), and rich factor (transcriptomic). In the enrichment scatter plot, TCS and PTS exhibited the most significant changes across both two omics datasets. Additionally, top pathways such as ABC transporter, purine metabolism, QS, starch and sucrose metabolism, alanine/aspartate/glutamate metabolism, and glyoxylate/dicarboxylate metabolism remained highly enriched. In these pathways, TCS, QS, and PTS were known to be involved in biofilm formation. Therefore, the DEMs and DEGs regulated in the three pathways were further screened.

In the regulatory network diagram of the screened DEGs and DEMs, blue lines represent QS, orange lines indicate TCS, and purple lines depict the PTS pathway (Figure 5D). These pathways play a crucial role in regulating metabolism. Norepinephrine was down-regulated by the *Rgg/GadR/MutR* family transcriptional regulators *rgg3* and *rgg2*. The response regulator transcription factors *arlR*, *vicR*, *ciaR*, *vicK*, and *gtfS* down-regulated L-glutamic acid, L-glutamine, and L-aspartic acid expression levels while up-regulating succinic acid, L-malic acid, and citric acid expression levels. Additionally, four subunits of sugar transporters were down-regulated along with N-acetyl-D-glucosamine. Conversely, PTS mediated the up-regulation of phosphoenolpyruvic acid and cellobiose.

Pearson correlation analysis was used to explore the relationship between metabolites and biofilm characteristics (Figure 5E). The green line represents the most significant characteristic, blue lines depict other noteworthy features, and yellow lines indicate insignificance. All investigated characteristics demonstrated a substantial association with metabolites. For instance, phosphoenolpyruvic acid and cellobiose were highly related to viability. L-aspartic acid significantly related to WSG and hydrophobicity. L-glutamine was related to adhesion.

While phosphoenolpyruvic acid and cellobiose were down-regulated by the sugar transporter protein in the PTS pathway, viability was down-regulated by phosphoenolpyruvic acid and cellobiose (Figures 5D,E). After L-aspartic acid was regulated by the response regulator transcription factor, cell wall metabolism sensor histidine, and glycosyltransferase in TCS, WSG and hydrophobicity were then down-regulated. Moreover, adhesion was down-regulated by L-glutamine through its regulation via TCS. WIG was down-regulated by PTS-mediated regulation of phosphoenolpyruvic acid. *Rgg/GadR/MutR* family transcriptional regulators were associated with norepinephrine through the QS signaling pathway, which had a relationship with viability, WSG, and hydrophobicity. Rgg/GadR/MutR family transcriptional regulators was an important part of TCS. In all, TCS was the central pathway regulated by LJF.

**Figure 6:**
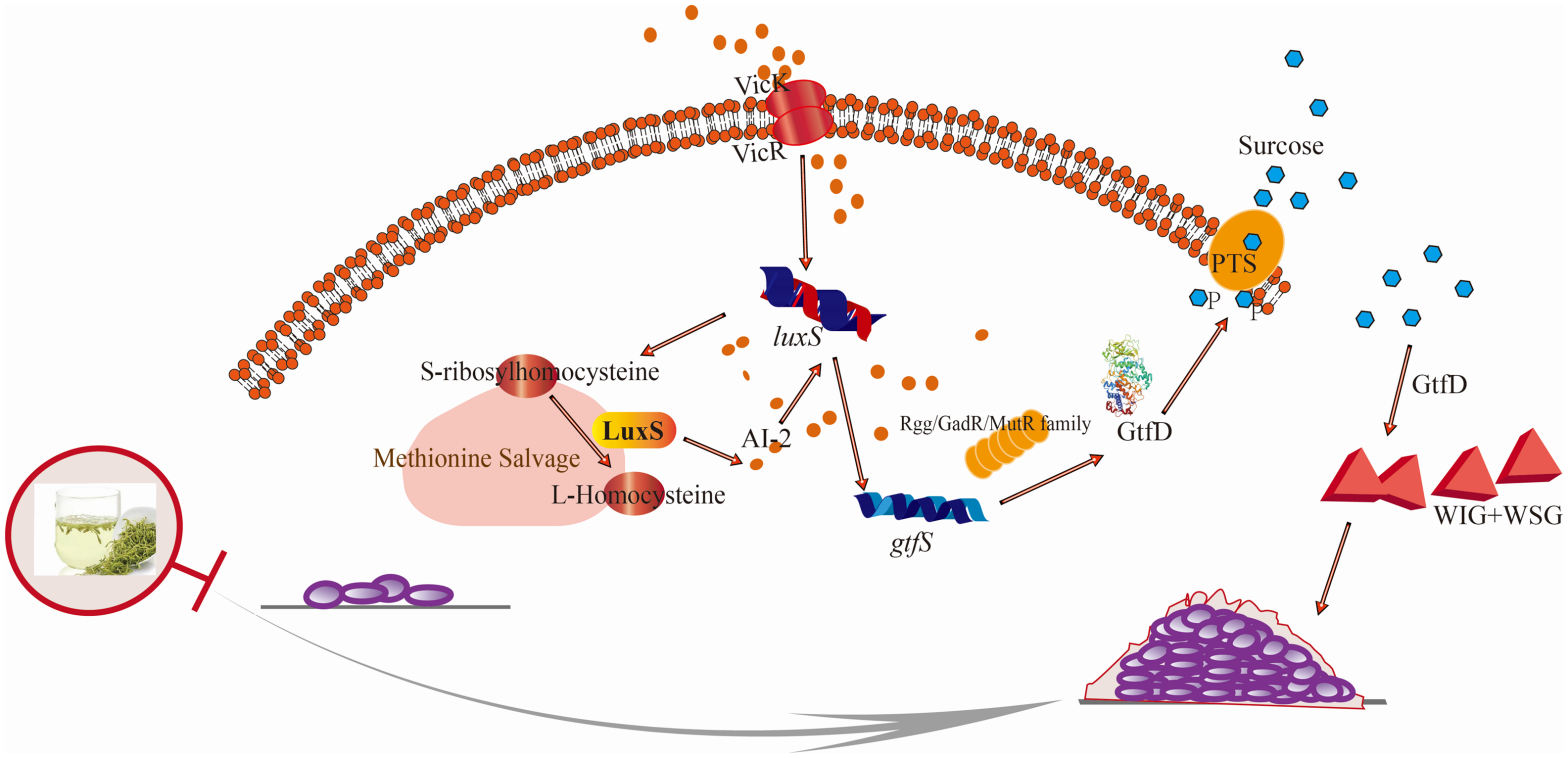
Schematic illustration of the anti-biofilm mechanism of LJF against *S. mutans*. Briefly, the sensing histidine VicK was expressed disorderly and AI-2 sensing was blocked by LJF. The corresponding response regulator transcriptional factor was inhibited, leading to the down-regulated glycosyltransferase by gtf family and decreased in WIG and WSG.

Interestingly, genes of *gtfS* and *vicK* were crucial within TCS, validating their role in this mechanism. Both genes exhibited down-regulation upon exposure to LJF (Figure 5F). The observed changes in *gtfS* expression suggested that LJF influenced *Gtf* activity while decreasing WIG and WSG production. Alterations in *vicK* expression provided evidence for TCS-mediated biofilm inhibition under LJF-induced stress conditions (see Figure 6).

## 4 Discussion

Until the 2005 Edition of Chinese patent medicines, LJF (Henan and Shandong in Figure 1) and *Lonicerae flos* (LF, Hunan and Guizhou in Figure 1) were long considered the same herb. Relevant pharmacological distinctions between them have been scarcely investigated. From previous study, LJF and LF have similar antibacterial spectrums (Li Y. et al., 2020). But in this study, LJF exhibited stronger antibacterial activity than LF. The origin of LJF and LF used in our study differs from previous studies, leading to different results. Studies showed that the antibacterial activity was highly correlated with the phenolic acid content and phenolic acid content can be reflected by lower chromatic value (Shan et al., 2007; Gao et al., 2021). As indicated in Supplementary Table S1, the chromatic value of LJF was lower than that of LF. It means the content of phenolic acid in LJF was higher than that in LF. From Figure 1B, LJF has the more anti-biofilm activity than LF. Therefore, the content of phenolic acid in LJF may also related to the inhibitory effect of biofilm formation. In recent years, there are few studies focus on plant extracts’ inhibitory effect of biofilm formation. *Cymbopogon citratus* displayed the highest efficacy in reducing *S. mutans* biofilm formation and adhesion activity, achieving 90% inhibition at an MIC value of 12 μg/mL (Preeti Pallavi, 2024). However, the LJF has inhibitory effect at more than 6.00 mg/mL in our study. Maybe it caused by the different methods of extracts preparation. Different methods of extracts preparation leaded to difference phytochemicals and functionality in extracts.

The mechanisms of antibacterial activity and anti-biofilm formation are distinct. Antibacterial activity involves cell wall synthesis suppression, membrane permeability alteration, protein synthesis inhibition, nucleic acid metabolism perturbation, and folate metabolism modulation (Silva et al., 2016). The anti-biofilm mechanisms are less understood, focusing on quorum sensing inhibition, adhesion protein disruption, and second messenger pathways (Koo et al., 2017). Biofilms are complex, including bacteria, metabolites, polysaccharide matrix, fibrin, and lipids, and are susceptible to environmental influences (Yadav et al., 2020; Rather et al., 2021). Polysaccharide matrix was the main component of biofilm. EPS was the polysaccharide matrix outside bacteria cells. The components of EPS varied among different bacteria. For *S. mutans*, the main constituent of EPS is predominantly glucan. WIG and WSG are crucial for biofilm formation and resistance to environmental factors, with their reduction consistent with observed inhibitory effect of biofilm formation. In biofilm formation, *S. mutans* adheres to *in situ* glucans formed by Gtfs and glucan-binding proteins (Gbps). Gtfs hydrolyze sucrose, with *S. mutans* possessing multiple Gbps (GbpA, GbpB, GbpC, and GbpD) facilitating adhesion (Bhatt et al., 2020). WIG, rich in α-1,3-glucosidic linkages, is synthesized by GtfB and GtfC and plays a crucial role in biofilm accumulation and adhesion, while GtfD synthesizes WSG rich in α-1,6-glucosidic linkages (Matsumoto-Nakano, 2018; Zhang H. et al., 2022). Reduced Cellular aggregation reflects inter-bacterial interaction, with stronger aggregation indicating higher invasiveness, driven by extracellular glucan (Vickerman and Jones, 1995; Yi Wang, 2014). Cell surface hydrophobicity, influenced by dextran-bound hydrophobic moieties, affects biofilm formation, with reduced hydrophobicity indicating modified dextran properties (Schormann et al., 2023; Zheng et al., 2023). In LJF, not only the WSG and WIG were down-regulated (Figures 2H,I), but also the expression level of *gtfS* was inhibited. It proposed that the down-regulated in WIG and WSG production indicated the decreased Gtf activity. LJF disrupted the EPS-mediated adherence pathway, impacting biofilm surface properties.

AI-2, a ubiquitous signaling molecule, plays a crucial role in various metabolic pathways and the LuxS/AI-2 QS system in *S. mutans*, which governs drug resistance, biofilm formation, motility, adherence, and virulence (Zhang et al., 2019). AI-2, produced through the LuxS-catalyzed S-ribosyl homocysteine (SRH) pathway, decreased in the presence of LJF, confirming its inhibitory effect on biofilm formation. The AI-2 inhibition mechanism involves obstructing the histidine kinase VicK or using LJF components as AI-2 analogs (Li et al., 2021; Yuan et al., 2021; Zhang Y. et al., 2022). Despite many studies identifying anti-biofilm inhibitors, understanding the biofilm inhibition mechanism is limited. The crystal violet assay, transcriptome analysis, PCR, and microstructure analyses reveal phytochemicals’ potential in combating biofilm formation, but their mechanisms remain elusive.

Untargeted metabolomics analyses using LC–MS can identify new biomarkers and diagnostics, with bacterial metabolites identified as lipids, amino sugars, nucleotides, amines, organic acids, peptides, amino acids, and terpenes (Depke et al., 2020; Zhao et al., 2023; Bernardo-Bermejo et al., 2024). Although there are many bacterial metabolites identified, reports on biofilm bacteria metabolites are scarce. Maytham Hussein pioneered the application of a non-targeted metabolomics approach to elucidate the mechanism of action of Texobatine against methicillin-resistant *S. aureus* (Hussein et al., 2020). A total of 6,209 differential metabolites can be screened to explore the mechanism of lipoic acid against *Y. enterocolitica* (Yang et al., 2022). It is valuable to for understanding these mechanisms. In fact, to the best of our knowledge, few studies have been conducted on *S. mutan*’s metabolomics based on LC–MS. The current research on the anti-biofilm mechanism is still limited. As many regulators and effectors of virulence are small-molecule secondary metabolites that can be generally amenable to LC–MS (Letieri et al., 2022), the development of an untargeted metabolomics approach based on LC–MS for comprehensive analysis of the known metabolome at a large scale would be highly valuable in elucidating the molecular mechanisms underlying anti-biofilm activity. In this study, under LJF stress, fatty acyls, carboxylic acids, amino acids, steroids, prenol lipids, and benzene derivatives were the main DEMs (Figure 3). Amino acids, serving as energy precursors, indicated high metabolic activity and biofilm maturity under LJF stress (Sadiq et al., 2020). Biofilm development is energy-intensive, with histidine and alanine metabolism crucial for *E. coli* and *S. mutans* biofilm formation, respectively. Methionine metabolism also plays a role in biofilm structure phenotype by regulating the LuxS QS system in *S. mutans* (Hu et al., 2018).

In response to LJF, all the DEGs attributed in peptidoglycan biosynthesis and terpenoid backbone biosynthesis were down-regulated. Peptidoglycan is a crucial component of the cell wall, consisting of peptide and glycan molecules (Sara Marti, 2017). Peptidoglycan enhances bacterial resistance to stressful environments, while down-regulating its synthesis pathway can reduce bacteria’s adaptability to their surroundings (Christopher Ealand and Germar Beukes, 2024). It means that the inhibitory effect of LJF on decreasing bacterial resistance prevented drug resistance. Terpenoid backbone biosynthesis in bacteria is a complex biochemical process that involves the synthesis of terpene precursors and their subsequent modification to form various terpenoid compounds. But the biosynthesis of terpenoids remains unclear (Duan et al., 2023). LJF down-regulated DEGs involved in peptidoglycan and terpenoid backbone biosynthesis, reducing bacterial resistance and adaptability. Dextransucrase, down-regulated by LJF, inhibited dextran synthesis, the main WIG in EPS (Fujiwara et al., 1998). From the decreased WIG production, it can be inferred that the activity of dextransucrase may also inhibited by LJF. Levanbiose, the main WSG, was produced through the enzymatic conversion of sucrose by levansucrase, followed by levanase-catalyzed formation (Pereira et al., 2001). LJF impeded the activity of glycosyltransferase, reducing glucan production. *S. mutans* has two sucrose metabolic pathways: extracellular Gtf metabolism and intracellular PTS transporter, with extracellular Gtf activity being the primary biofilm formation mechanism. LJF inhibited Gtf expression (Figure 5F), suppressing Gtf activity through the Rgg/GadR/MutR family transcriptional regulator and the QS system (Supplementary Figure S3). Conjoint analysis revealed that WSG is influenced by the QS system (Figures 5D,E). Decreased expression of *rgg3* and *rgg2*, were validated by RT-PCR (Supplementary Figure S3). It indicated that the impact of LJF on Gtf activity was through TCS. The expression of the histidine kinase VicK (Supplementary Table S5) was disrupted and AI-2 sensing was blocked (Figure 2K), hindering response regulator transcription factors (Supplementary Figure S3), resulting in decreased Gtf activity and reduced WIG and WSG production (Figures 2H,I). L-aspartic acid and norepinephrine, regulated by TCS and QS, influenced WSG. Phosphoenolpyruvic acid, inhibited via PTS and regulated WIG (Figures 5D,E). In summary, LJF primarily inhibited biofilm formation by suppressing glucan production through the QS system, TCS, and PTS. This inhibition was triggered by LJF disrupting the expression of histidine kinase VicK and blocking AI-2 sensing, leading to the down-regulation of Gtf activity and a decrease in both WIG and WSG. For LJF, the primary targets of inhibitory effects on biofilm formation were VicK and Gtf.

## 5 Conclusion

*Lonicera japonica flos* (LJF) significantly reduced EPS production within the biofilm, accounting for its inhibitory activity. Mechanistically, WIG and WSG were down-regulated by Gtf, associated with TCS, QS, and PTS pathways, leading to the reduced EPS production. WSG synthesis was regulated by L-aspartic acid and norepinephrine via TCS and QS pathways, respectively, while WIG synthesis was regulated by phosphoenolpyruvic acid via the PTS pathway. The regulation trigger was that LJF disrupted VicK and blocked AI-2 sensing, inhibiting response regulator transcriptional factors. This pioneering study on LJF’s inhibitory mechanism against *S. mutans* provides a solid foundation for developing LJF-based functional food and oral health care products.

## Data availability statement

In the part of 2.9, the RNA-seq datasets are available at the NCBI Sequence Read Archive (SRA) database under accession number PRJNA1119033. The complete dataset could be accessed at https://www.ncbi.nlm.nih.gov/sra/PRJNA1119033. In the part of 2.8, Metabolomics data have been deposited to the EMBL-EBI Metabolites database: with the identifier MTBLS9711. The complete dataset could be accessed at https://www.ebi.ac.uk/metabolights/MTBLS9711.

## Author contributions

LW: Conceptualization, Investigation, Methodology, Writing – original draft, Writing – review & editing. PL: Data curation, Investigation, Writing – review & editing. YW: Data curation, Writing – review & editing. HP: Investigation, Writing – review & editing. XC: Methodology, Funding acquisition, Resources, Writing – review & editing, Supervision, Project administration.

## Footnotes

^1^ https://www.biodeep.cn/

## Glossary

LJF: *Lonicera japonica* flos
*S. mutans*: *Streptococcus mutans*
RT-PCR: Real-time fluorescent quantitative Polymerase Chain Reaction
EPS: Exopolysaccharides
CH: Chlorhexidine
BHI: Brain-heart infusion
BHIS: Brain-heart infusion containing 1% sucrose
MIC: minimum inhibitory concentration
CV: crystal violet
SEM: scanning electron microscopy
FTIR: Fourier transform infrared spectroscopy
WIG: Water insoluble glucan
WSG: Water soluble glucan
AC: Aggregation capacity
RLI: Relative luminescence intensity
UPLC: Ultrahigh-performance liquid chromatography
MS: Mass spectrometry
VIP: Variable Important in projection
FDR: False discovery rate
TCS: two-component system
QS: quorum sensing system
PTS: phosphotransferase system
