# Supplementary material for: Inhibitory effect of Lonicera japonica flos on Streptococcus mutans biofilm and mechanism exploration through metabolomic and transcriptomic analyses
Source: Front Microbiol. 2024 Jul 3;15:1435503. doi: 10.3389/fmicb.2024.1435503 (PMC11256199; doi:10.3389/fmicb.2024.1435503)
Supplement: Supplementary file 1 [file Data_Sheet_1.docx]

Supplementary Material

# **Supplementary Figures and Tables**

## **Supplementary Figures**

**
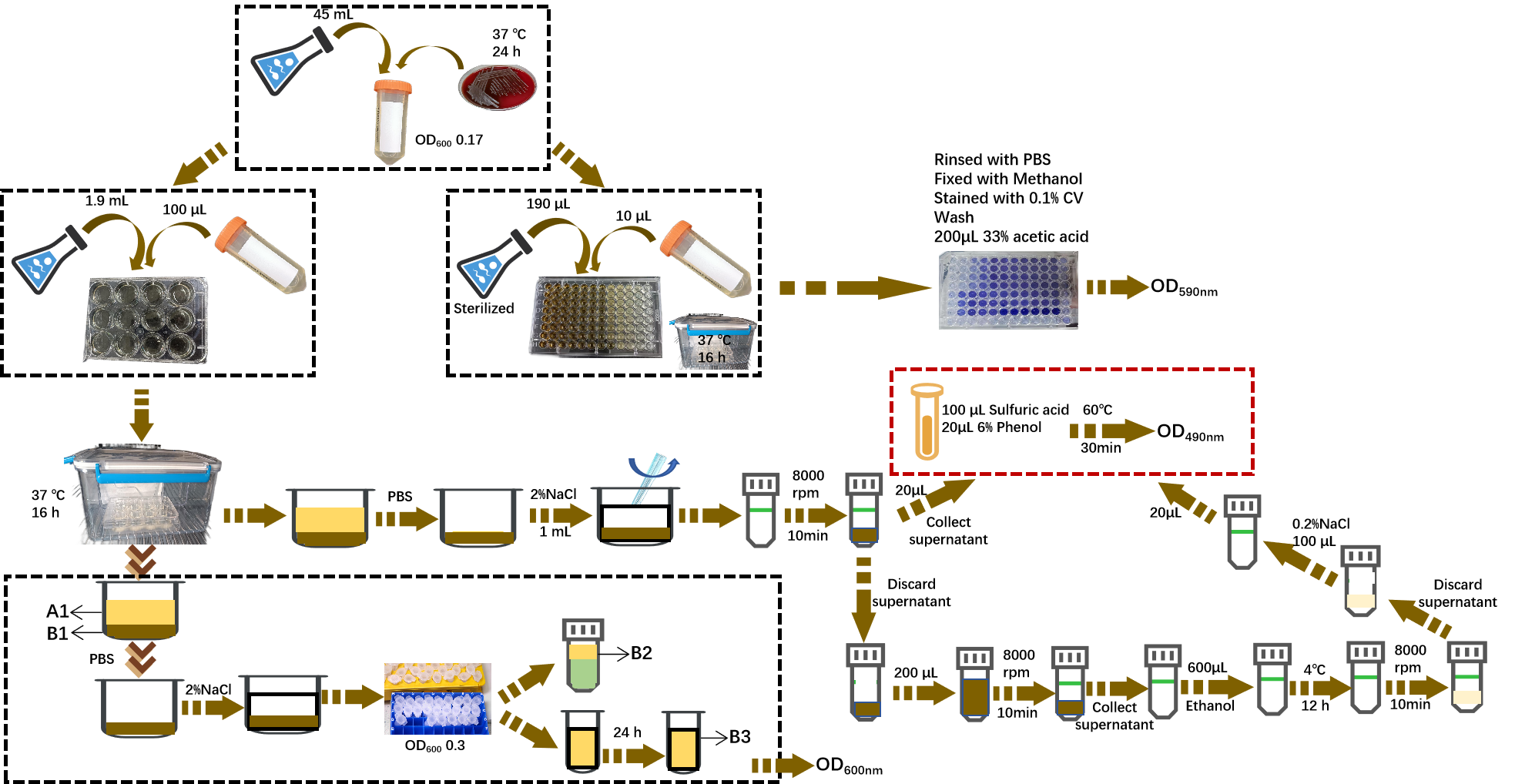
**

**Supplementary Figure 1.** Detailed steps for determination of biofilm formation, surface character, water-soluble glucan and water-insoluble glucan.

**
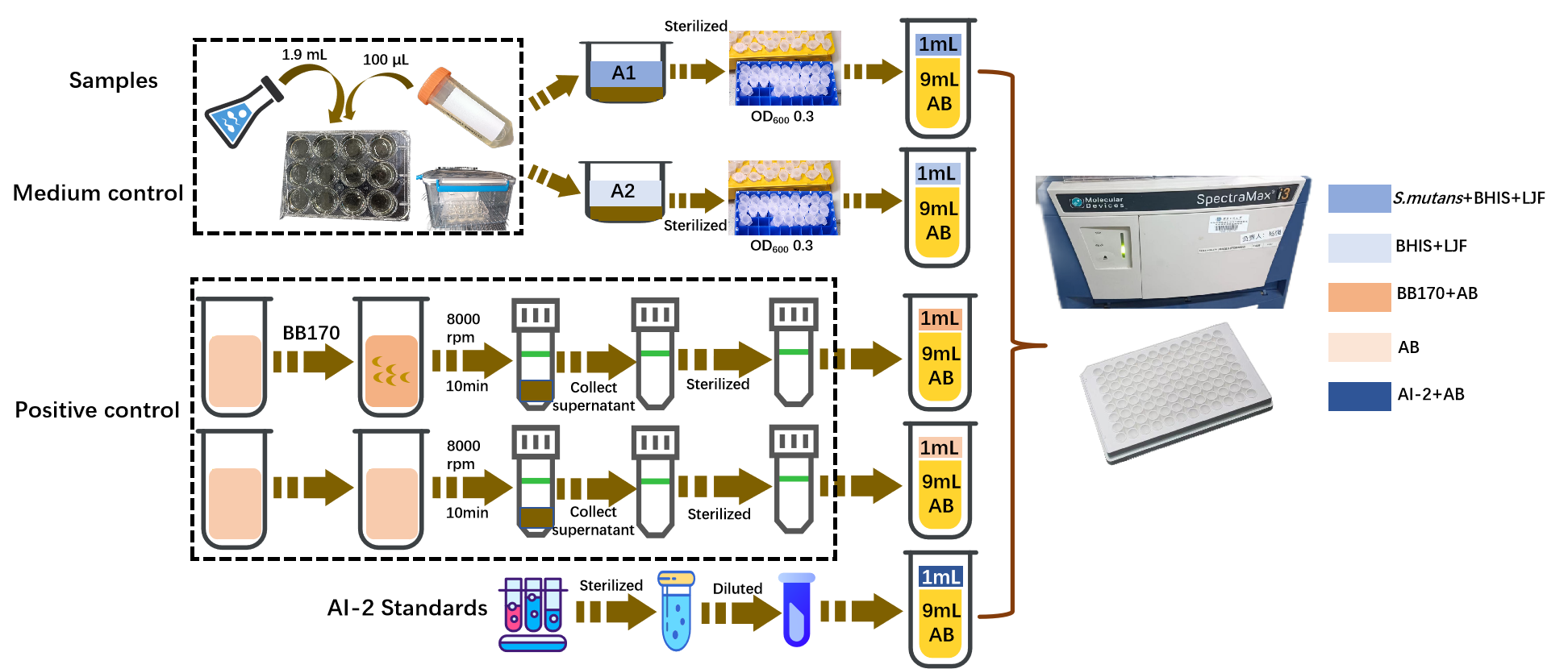
**

**Figure S2.** Detailed steps for determination of AI-2

**Table S1.** Sample Information

|  | HENAN | SHANDONG | HUNAN | GUIZHOU |
| --- | --- | --- | --- | --- |
| Species | *Lonicera japonica Thunberg* | *Lonicera japonica Thunberg* | *Lonicera macranthoides* | *Lonicera macranthoides* |
| Origin | Fengqiu, Henan, Chana | Pingyi, Shandong, China | Longhui, Hunan, China | Suiyang, Guizhou, China |
| Collection Date | 2021.6 | 2021.6 | 2021.8 | 2021.7 |
| Chromatic Value | L: 53.65 | L: 51.65 | L: 66.09 | L:57.7 |
|  | a: 2.08 | a: 3.02 | a: 3.20 | a: 2.77 |
|  | b: 27.39 | b: 27.32 | b: 33.86 | b: 29.52 |
|  | c: 27.47 | c: 27.48 | c: 34.01 | c: 29.65 |
|  | h: 85.66 | h: 83.69 | h: 84.61 | h: 84.65 |

L: light and shade; a: red-green value; b: yellow and blue value; c: color saturation; h: hue.

**Table S2.** Primers of genes for RT-PCR

| Description | F | R |
| --- | --- | --- |
| *oppA* | AGTAATCTGTTGCGTGTT | CAATGTTGCTGTGTAAGTTA |
| *gtf-S* | ACCAGTCTTGTAGTTGATAG | CAGCAGTTGTTACCATTG |
| *ftsA* | ACCAGTCTTGTAGTTGATAG | CAGCAGTTGTTACCATTG |
| *glnA* | GGTAATCCGACAACAGAAGTAA | GGTATTATCAGCAAGGTCAGTT |
| *arlR* | ATGAAGGATATGAGGTTGAAG | ACGAGCAGTGATAATAATAACA |
| *ciaH* | CAGAAGAACAGGCAGAGT | ACGAGTAGCATCCAAGAC |
| *sacB* | AACGGCGACTTACTCTTAT | AACGGCGACTTACTCTTAT |
| *vicR* | GTGGAACAGAGGTAGAGT | ACGAACAGTAACATCAACA |
| *ciaH2* | AATGTCAATGCCTTCTCTTC | ACTGTCACTGTCCGATAC |
| *braD* | TTTATTAGAAAAGCGTCCCT | TGTTGGTTCATCTGCTAAAA |
| *vicK* | GAACTGCGGACACCATTA | TAGGAGCCACTGATTCTGT |
| *lrgB* | AATCTGTTACAACCGCAATG | GTCCAGTTCCACCAAGAG |
| *comX1_2* | CCAGAATTAGAAGAGGATGATACA | CATACCGCCACTTGACAA |
| *rgg2* | TATTTCACGTATGGCTCTGA | TGAAAGACCAAGCGTTCATA |
| *rgg3* | TGAAGAGTGGACAGAATATGAA | TGGAATGGACAAGTATAATGAATC |
| *16S* | CTCAGACACAGGTGCTGCAT | CACCGGCAGTCTCCTTAGAG |

oppA: SMU_RS01295; gtf-S: SMU_RS04210; ftsA: SMU_RS03590; glnA: SMU_RS01795; arlR: SMU_RS08745; ciaH: SMU_RS05195; sacB: SMU_RS09270; vicR: SMU_RS06885; ciaH2: SMU_RS04285; braD: SMU_RS04630; vicK: SMU_RS06880; lrgB: SMU_RS07720; comX1_2: SMU_RS09085; rgg2: SMU_RS06845; rgg3: SMU_RS00580.

**Table S3.** Primers of genes for RT-PCR

| Sample | Raw reads | Raw bases(G) | Clean reads | Clean bases(G) | Q20(%) | Q30(%) | GC content(%) | Clean data ratio(%) |
| --- | --- | --- | --- | --- | --- | --- | --- | --- |
| BHIS_1 | 20319470 | 3.05 | 17254954 | 2.5 | 98.01 | 93.29 | 36.09 | 81.94 |
| BHIS_2 | 18985146 | 2.85 | 15996962 | 2.31 | 97.95 | 93.16 | 35.97 | 81.26 |
| BHIS_3 | 20367674 | 3.06 | 17159616 | 2.48 | 97.95 | 93.15 | 36.68 | 81.29 |
| LJF_1 | 19894724 | 2.98 | 17755698 | 2.61 | 98.24 | 93.78 | 38.01 | 87.41 |
| LJF_2 | 17287912 | 2.59 | 15522224 | 2.28 | 98.32 | 93.99 | 38 | 88.03 |
| LJF_3 | 13645846 | 2.05 | 12073926 | 1.77 | 98.14 | 93.61 | 37.61 | 86.69 |

**Table S4.** Identified DEMs

| Compound Name | RT_Exact Mass | MS Error (ppm) | Formula | Adducts | Classification | CAS No. |
| --- | --- | --- | --- | --- | --- | --- |
| Cyanuric acid | 9.98_128.0083 | 14.2491 | C3H3N3O3 | [M-H]- | Triazines | 108-80-5 |
| 17a-Estradiol | 6.97_255.1744 | 16.5926 | C18H24O2 | [M+H-H2O]+ | Steroids and steroid derivatives | 57-91-0 |
| Androsterone | 7.74_291.1945 | 6.0391 | C19H30O2 | [M+H]+ | Steroids and steroid derivatives | 53-41-8 |
| Pregnanediol | 11.25_321.3146 | 1.4842 | C21H36O2 | [M+H]+ | Steroids and steroid derivatives | 80-92-2 |
| Corticosterone | 6.52_347.2212 | 1.3709 | C21H30O4 | [M+H]+ | Steroids and steroid derivatives | 50-22-6 |
| Cholesterol | 10.73_369.3499 | 5.9564 | C27H46O | [M+H-H2O]+ | Steroids and steroid derivatives | 57-88-5 |
| Desmosterol | 7.29_384.3417 | 6.5046 | C27H44O | [M]+ | Steroids and steroid derivatives | 313-04-2 |
| Vitamin D3 | 9.90_385.2912 | 6.5046 | C27H44O | [M+H]+ | Steroids and steroid derivatives | 67-97-0 |
| Allocholic acid | 10.90_408.3681 | 1.7910 | C24H40O5 | [M]+ | Steroids and steroid derivatives | 2464-18-8 |
| Ursodeoxycholic acid | 10.10_393.2961 | 9.8552 | C24H40O4 | [M+H]+ | Steroids and steroid derivatives | 128-13-2 |
| 6beta-Hydroxytestosterone | 8.76_287.2000 | 12.6532 | C19H28O3 | [M+H-H2O]+ | Steroids and steroid derivatives | 62-99-7 |
| Etiocholanolone | 9.34_291.2317 | 6.0391 | C19H30O2 | [M+H]+ | Steroids and steroid derivatives | 53-42-9 |
| Medroxyprogesterone | 10.26_345.2371 | 15.2823 | C22H32O3 | [M+H]+ | Steroids and steroid derivatives | 520-85-4 |
| alpha-Spinasterol | 10.71_395.3643 | 3.1212 | C29H48O | [M+H-H2O]+ | Steroids and steroid derivatives | 481-18-5 |
| Stigmasterol | 11.33_395.3653 | 3.1212 | C29H48O | [M+H-H2O]+ | Steroids and steroid derivatives | 83-48-7 |
| beta-Sitosterol | 11.31_397.3860 | 6.5428 | C29H50O | [M+H-H2O]+ | Steroids and steroid derivatives | 83-46-5 |
| Brassinolide | 9.93_481.3492 | 6.5981 | C28H48O6 | [M+H]+ | Steroids and steroid derivatives | 72962-43-7 |
| 5alpha-Cholestanone | 9.40_369.3526 | 5.9564 | C27H46O | [M+H-H2O]+ | Steroids and steroid derivatives | 566-88-1 |
| Clionasterol | 10.04_397.3862 | 6.5428 | C29H50O | [M+H-H2O]+ | Steroids and steroid derivatives | 83-47-6 |
| Nandrolone | 8.50_275.2004 | 0.6395 | C18H26O2 | [M+H]+ | Steroids and steroid derivatives | 434-22-0 |
| 20-Hydroxyecdysone | 7.56_463.3048 | 7.6278 | C27H44O7 | [M+H-H2O]+ | Steroids and steroid derivatives | 5289-74-7 |
| 17alpha,21-Dihydroxypregnenolone | 8.00_349.2371 | 0.7903 | C21H32O4 | [M+H]+ | Steroids and steroid derivatives | 1167-48-2 |
| 5a-Cholest-8-en-3b-ol | 8.43_369.3458 | 5.9564 | C27H46O | [M+H-H2O]+ | Steroids and steroid derivatives | 566-97-2 |
| Lathosterol | 11.22_369.3493 | 5.9564 | C27H46O | [M+H-H2O]+ | Steroids and steroid derivatives | 80-99-9 |
| 7-Dehydrodesmosterol | 9.85_383.3130 | 5.2747 | C27H42O | [M+H]+ | Steroids and steroid derivatives | 1715-86-2 |
| 5-Dehydroavenasterol | 10.75_393.3507 | 8.2217 | C29H46O | [M+H-H2O]+ | Steroids and steroid derivatives | / |
| Mifepristone | 6.11_430.2803 | 14.4650 | C29H35NO2 | [M+H]+ | Steroids and steroid derivatives | 84371-65-3 |
| Dehydroepiandrosterone | 11.37_269.2111 | 4.1088 | C19H28O2 | [M-H2O-H]- | Steroids and steroid derivatives | 53-43-0 |
| Deoxycholic acid | 6.67_391.2863 | 9.8552 | C24H40O4 | [M-H]- | Steroids and steroid derivatives | 83-44-3 |
| Chenodeoxycholic acid | 7.67_391.2861 | 9.8552 | C24H40O4 | [M-H]- | Steroids and steroid derivatives | 474-25-9 |
| Cholesterol sulfate | 9.77_465.3035 | 1.9342 | C27H46O4S | [M-H]- | Steroids and steroid derivatives | 1256-86-6 |
| Sphingosine 1-phosphate | 7.56_378.2410 | 1.3219 | C18H38NO5P | [M-H]- | Sphingolipids | 26993-30-6 |
| Pyrrole-2-carboxylic acid | 9.75_111.0201 | 1.2681 | C5H5NO2 | [M]+ | Pyrroles | 634-97-9 |
| UDP | 0.99_405.0083 | 1.6026 | C9H14N2O12P2 | [M+H]+ | Pyrimidine nucleotides | 58-98-0 |
| UMP | 0.76_323.0279 | 2.2413 | C9H13N2O9P | [M-H]- | Pyrimidine nucleotides | 58-97-9 |
| CMP | 0.76_322.0431 | 4.6183 | C9H14N3O8P | [M-H]- | Pyrimidine nucleotides | 63-37-6 |
| CDP | 0.71_402.0106 | 0.3537 | C9H15N3O11P2 | [M-H]- | Pyrimidine nucleotides | 63-38-7 |
| Deoxyuridine | 10.84_228.1957 | 0.0190 | C9H12N2O5 | [M]+ | Pyrimidine nucleosides | 951-78-0 |
| Thymidine | 11.36_223.0278 | 0.4142 | C10H14N2O5 | [M-H2O-H]- | Pyrimidine nucleosides | 50-89-5 |
| Uridine | 6.12_243.0667 | 18.4147 | C9H12N2O6 | [M-H]- | Pyrimidine nucleosides | 58-96-8 |
| Nornicotine | 4.51_149.1075 | 1.5023 | C9H12N2 | [M+H]+ | Pyridines and derivatives | 494-97-3 |
| 4-Pyridoxic acid | 0.62_182.9834 | 10.7023 | C8H9NO4 | [M]+ | Pyridines and derivatives | 82-82-6 |
| Niacinamide | 2.45_123.0563 | 8.1264 | C6H6N2O | [M+H]+ | Pyridines and derivatives | 98-92-0 |
| Nicotine | 3.51_163.1232 | 3.1714 | C10H14N2 | [M+H]+ | Pyridines and derivatives | 494-97-3 |
| Pyridoxamine | 1.22_169.0973 | 0.7333 | C8H12N2O2 | [M+H]+ | Pyridines and derivatives | 85-87-0 |
| ADP | 1.02_428.0357 | 2.2802 | C10H15N5O10P2 | [M+H]+ | Purine nucleotides | 58-64-0 |
| Adenosine diphosphate ribose | 1.04_542.0646 | 0.6162 | C15H23N5O14P2 | [M+H-H2O]+ | Purine nucleotides | 20762-30-5 |
| dAMP | 1.16_330.0613 | 1.1392 | C10H14N5O6P | [M-H]- | Purine nucleotides | 653-63-4 |
| AMP | 0.82_346.0546 | 3.4677 | C10H14N5O7P | [M-H]- | Purine nucleotides | 61-19-8 |
| GMP | 0.77_362.0485 | 6.0765 | C10H14N5O8P | [M-H]- | Purine nucleotides | 85-32-5 |
| Adenylsuccinic acid | 0.66_462.0673 | 1.0821 | C14H18N5O11P | [M-H]- | Purine nucleotides | 19046-78-7 |
| Adenosine | 0.94_268.1032 | 2.9839 | C10H13N5O4 | [M+H]+ | Purine nucleosides | 58-61-7 |
| Guanosine | 1.22_282.0831 | 3.5697 | C10H13N5O5 | [M-H]- | Purine nucleosides | 118-00-3 |
| Inosine | 0.94_267.0724 | 4.1187 | C10H12N4O5 | [M-H]- | Purine nucleosides | 58-63-9 |
| Tetrahydropteridine | 1.85_136.0762 | 9.5535 | C6H8N4 | [M]+ | Pteridines and derivatives | 10593-78-9 |
| 7,8-Dihydro-beta-carotene | 8.40_521.4610 | 27.8725 | C40H58 | [M+H-H2O]+ | Prenol lipids | / |
| Perillyl alcohol | 8.24_135.1169 | 3.7005 | C10H16O | [M+H-H2O]+ | Prenol lipids | 536-59-4 |
| Maslinic acid | 8.61_455.3487 | 1.8316 | C30H48O4 | [M+H-H2O]+ | Prenol lipids | 4373-41-5 |
| 2-trans,6-trans-Farnesal | 8.92_203.1793 | 19.8544 | C15H24O | [M+H-H2O]+ | Prenol lipids | 502-67-0 |
| Capsidiol | 7.98_219.1744 | 2.2813 | C15H24O2 | [M+H-H2O]+ | Prenol lipids | 37208-05-2 |
| (S)-2,3-Epoxysqualene | 11.42_409.3816 | 6.4341 | C30H50O | [M+H-H2O]+ | Prenol lipids | 54910-48-4 |
| Lutein | 9.26_569.4191 | 28.4079 | C40H56O2 | [M+H]+ | Prenol lipids | 127-40-2 |
| Curcumenol | 6.17_235.1688 | 0.4746 | C15H22O2 | [M+H]+ | Prenol lipids | 19431-84-6 |
| Nootkatone | 9.91_219.1743 | 0.3468 | C15H22O | [M+H]+ | Prenol lipids | 4674-50-4 |
| Zerumbone | 7.37_219.1744 | 0.3468 | C15H22O | [M+H]+ | Prenol lipids | 471-05-6 |
| Ursolic acid | 10.37_439.3575 | 10.5472 | C30H48O3 | [M+H-H2O]+ | Prenol lipids | 77-52-1 |
| Glycyrrhetinate | 8.53_453.3355 | 7.3544 | C30H46O4 | [M+H-H2O]+ | Prenol lipids | 1449-05-4 |
| Perillyl aldehyde | 5.34_133.1009 | 1.7409 | C10H14O | [M+H-H2O]+ | Prenol lipids | 2111-75-3 |
| Cuminaldehyde | 8.51_149.0961 | 0.0985 | C10H12O | [M+H]+ | Prenol lipids | 122-03-2 |
| Carnosol | 6.97_331.1880 | 0.0203 | C20H26O4 | [M+H]+ | Prenol lipids | 5957-80-2 |
| 4a-Carboxy-4b-methyl-5a-cholesta-8,24-dien-3b-ol | 10.66_425.3252 | 28.3689 | C29H46O3 | [M+H-H2O]+ | Prenol lipids | / |
| Taraxerol | 9.02_427.3560 | 6.4341 | C30H50O | [M+H]+ | Prenol lipids | 127-22-0 |
| Lanosterin | 8.77_425.2545 | 6.4341 | C30H50O | [M-H]- | Prenol lipids | 79-63-0 |
| all-trans-Retinoic acid | 9.38_300.2046 | 14.3236 | C20H28O2 | [M]- | Prenol lipids | 302-79-4 |
| Coenzyme Q10 | 10.95_862.6768 | 8.2302 | C59H90O4 | [M]- | Prenol lipids | 303-98-0 |
| 3-(2-Hydroxyphenyl)propanoic acid | 8.11_167.0701 | 0.6182 | C9H10O3 | [M+H]+ | Phenylpropanoic acids | 495-78-3 |
| m-Cresol | 9.72_109.0220 | 6.1874 | C7H8O | [M+H]+ | Phenols | 108-39-4 |
| Hydroquinone | 9.72_110.0201 | 4.5520 | C6H6O2 | [M]+ | Phenols | 123-31-9 |
| Tyrosol | 9.52_121.0650 | 0.3353 | C8H10O2 | [M+H-H2O]+ | Phenols | 501-94-0 |
| Norepinephrine | 9.67_169.9761 | 7.5156 | C8H11NO3 | [M+H]+ | Phenols | 51-41-2 |
| 3,4-Dihydroxyphenylglycol | 4.44_170.0601 | 12.9366 | C8H10O4 | [M]+ | Phenols | 28822-73-3 |
| Chavicol | 6.90_135.0805 | 0.0662 | C9H10O | [M+H]+ | Phenols | 501-92-8 |
| Vanillylmandelic acid | 6.63_197.1176 | 1.0037 | C9H10O5 | [M-H]- | Phenols | 55-10-7 |
| 4-Nitrophenol | 5.71_138.0189 | 5.2457 | C6H5NO3 | [M-H]- | Phenols | 100-02-7 |
| Venlafaxine | 5.62_278.2137 | 7.9939 | C17H27NO2 | [M+H]+ | Phenol ethers | 93413-69-5 |
| N,N-Diethyl-m-toluamide | 6.95_192.1383 | 0.1249 | C12H17NO | [M+H]+ | Pesticides/Herbicides | 134-62-3 |
| Coumafuryl | 6.45_297.0767 | 0.4174 | C17H14O5 | [M-H]- | Pesticides/Herbicides | 117-52-2 |
| beta-Alanyl-L-arginine | 1.39_245.1490 | 0.8158 | C9H19N5O3 | [M]+ | Peptidomimetics | / |
| 2-Heptanone | 3.67_113.9630 | 8.6136 | C7H14O | [M]+ | Organooxygen compounds | 110-43-0 |
| D-Ribose | 0.70_151.0357 | 4.9566 | C5H10O5 | [M+H]+ | Organooxygen compounds | 613-83-2 |
| D-Arabitol | 8.74_152.9939 | 3.9664 | C5H12O5 | [M+H]+ | Organooxygen compounds | 7643-75-6 |
| 2,3-Butanediol | 10.30_154.9915 | 7.3150 | C4H10O2S2 | [M+H]+ | Organooxygen compounds | 513-85-9 |
| Sorbitol | 4.53_183.0917 | 29.4731 | C6H14O6 | [M+H]+ | Organooxygen compounds | 50-70-4 |
| myo-Inositol | 11.34_181.0145 | 2.3832 | C6H12O6 | [M+H]+ | Organooxygen compounds | 87-89-8 |
| Fructose 1,6-bisphosphate | 0.89_322.9913 | 13.5296 | C6H14O12P2 | [M+H-H2O]+ | Organooxygen compounds | 488-69-7 |
| N-Acetylmuramate | 0.89_276.1071 | 12.4372 | C11H19NO8 | [M+H-H2O]+ | Organooxygen compounds | 61633-75-8 |
| Jasmone | 8.92_165.1273 | 0.4603 | C11H16O | [M+H]+ | Organooxygen compounds | 488-10-8 |
| Deoxyribose | 9.58_117.0549 | 0.0148 | C5H10O4 | [M+H-H2O]+ | Organooxygen compounds | 533-67-5 |
| Chlorogenic acid | 5.08_355.1009 | 0.9176 | C16H18O9 | [M+H]+ | Organooxygen compounds | 327-97-9 |
| D-Xylose | 9.14 _149.0080 | 4.9566 | C5H10O5 | [M-H]- | Organooxygen compounds | 58-86-6 |
| D-Mannose | 1.23_180.0649 | 2.3832 | C6H12O6 | [M]- | Organooxygen compounds | 3458-28-4 |
| Mannitol | 5.97_181.0697 | 29.4731 | C6H14O6 | [M-H]- | Organooxygen compounds | 69-65-8 |
| N-Acetyl-D-glucosamine | 8.74_221.1541 | 0.4417 | C8H15NO6 | [M]- | Organooxygen compounds | 7512-17-6 |
| Ribose 1,5-bisphosphate | 7.73_309.1728 | 4.3125 | C5H12O11P2 | [M-H]- | Organooxygen compounds | 14689-84-0 |
| alpha-D-Ribose 1-phosphate | 0.75_229.011 | 4.0347 | C5H11O8P | [M-H]- | Organooxygen compounds | 14075-00-4 |
| D-Mannose 1-phosphate | 0.74_259.0215 | 3.5673 | C6H13O9P | [M-H]- | Organooxygen compounds | 27251-84-9 |
| Spermidine | 0.68_146.1651 | 0.0367 | C7H19N3 | [M+H]+ | Organonitrogen compounds | 124-20-9 |
| L-Carnitine | 0.83_162.1117 | 4.0033 | C7H15NO3 | [M+H]+ | Organonitrogen compounds | 541-15-1 |
| Porphobilinogen | 6.28_226.1798 | 3.0107 | C10H14N2O4 | [M]+ | Organonitrogen compounds | 487-90-1 |
| Sphinganine | 7.26_302.3048 | 0.5459 | C18H39NO2 | [M+H]+ | Organonitrogen compounds | 764-22-7 |
| Acetylcholine | 0.86_146.1173 | 2.0531 | C7H16NO2 | [M+H]+ | Organonitrogen compounds | 51-84-3 |
| Phosphorylcholine | 0.83_184.0731 | 0.0990 | C5H15NO4P | [M+H]+ | Organonitrogen compounds | 3616-04-4 |
| Choline | 0.82_104.1073 | 1.9211 | C5H14NO | [M]+ | Organonitrogen compounds | 62-49-7 |
| Oleoylethanolamide | 10.07_326.3043 | 3.2975 | C20H39NO2 | [M+H]+ | Organonitrogen compounds | 111-58-0 |
| Sphingosine | 7.41_300.2898 | 3.2502 | C18H37NO2 | [M+H]+ | Organonitrogen compounds | 123-78-4 |
| Taurine | 0.86_126.0220 | 0.0157 | C2H7NO3S | [M+H]+ | Organic sulfonic acids and derivatives | 107-35-7 |
| O-Phosphoethanolamine | 1.04_141.9584 | 1.9668 | C2H8NO4P | [M+H]+ | Organic phosphoric acids and derivatives | 1071-23-4 |
| Acetylphosphate | 8.40_139.9874 | 3.6140 | C2H5O5P | [M]+ | Organic phosphoric acids and derivatives | 590-54-5 |
| Phosphoenolpyruvic acid | 0.70_166.9739 | 7.1868 | C3H5O6P | [M-H]- | Organic phosphoric acids and derivatives | 138-08-9 |
| Phosphonoacetate | 0.73_138.9787 | 3.6140 | C2H5O5P | [M-H]- | Organic phosphonic acids and derivatives | 4408-78-0 |
| N-Methylethanolaminium phosphate | 4.47_156.0399 | 13.3041 | C3H10NO4P | [M+H]+ | Organic oxides | / |
| 7a,12a-Dihydroxy-5b-cholestan-3-one | 9.21_401.3329 | 10.8786 | C27H46O3 | [M+H-H2O]+ | Organic oxides | / |
| Teasterone | 10.07_431.3509 | 7.0338 | C28H48O4 | [M+H-H2O]+ | Organic oxides | / |
| 2-Deoxycastasterone | 10.61_431.3550 | 7.0338 | C28H48O4 | [M+H-H2O]+ | Organic oxides | 87734-68-7 |
| D-Fructose 2,6-bisphosphate | 0.68_339.9914 | 13.5296 | C6H14O12P2 | [M]- | Organic oxides | 79082-92-1 |
| Sucrose 6'-phosphate | 0.74_421.0727 | 5.9942 | C12H23O14P | [M-H]- | Organic oxides | / |
| Alantolactone | 5.17_233.1536 | 0.1029 | C15H20O2 | [M+H]+ | Naphthofurans | 546-43-0 |
| epsilon-Caprolactone | 11.45_114.0665 | 14.0269 | C6H10O2 | [M]+ | Lactones | 502-44-3 |
| Neocnidilide | 7.52_177.1274 | 23.3391 | C12H18O2 | [M+H-H2O]+ | Lactones | 4567-33-3 |
| Epsilon-caprolactam | 3.57_114.0921 | 1.3681 | C6H11NO | [M+H]+ | Lactams | 105-60-2 |
| 2-Ketobutyric acid | 4.98_102.0908 | 8.1205 | C4H6O3 | [M]+ | Keto acids and derivatives | 600-18-0 |
| Ketoleucine | 9.76_131.5336 | 19.9899 | C6H10O3 | [M+H]+ | Keto acids and derivatives | 816-66-0 |
| Oxoglutaric acid | 0.69_146.0298 | 0.2174 | C5H6O5 | [M]+ | Keto acids and derivatives | 328-50-7 |
| Oxoadipic acid | 0.99_158.9774 | 1.5301 | C6H8O5 | [M-H]- | Keto acids and derivatives | 3184-35-8 |
| alpha-Ketoisovaleric acid | 0.75_115.0386 | 12.3785 | C5H8O3 | [M-H]- | Keto acids and derivatives | 759-05-7 |
| 1-Benzyl-1,2,3,4-tetrahydroisoquinoline | 6.94_223.1329 | 14.3412 | C16H17N | [M]+ | Isoquinolines and derivatives | 19716-56-4 |
| Genistein | 6.23_271.0599 | 5.7129 | C15H10O5 | [M+H]+ | Isoflavonoids | 446-72-0 |
| Glycitein | 5.79_285.0764 | 0.3623 | C16H12O5 | [M+H]+ | Isoflavonoids | 40957-83-3 |
| Genistin | 7.77_432.2382 | 0.9356 | C21H20O10 | [M]+ | Isoflavonoids | 529-59-9 |
| Daidzein | 5.71_255.0647 | 0.0055 | C15H10O4 | [M+H]+ | Isoflavonoids | 486-66-8 |
| Formononetin | 8.45_269.0807 | 0.2974 | C16H12O4 | [M+H]+ | Isoflavonoids | 485-72-3 |
| Tryptophanol | 3.87_144.0808 | 28.6922 | C10H11NO | [M+H-H2O]+ | Indoles and derivatives | 526-55-6 |
| L-Tryptophan | 8.79_204.1377 | 5.9844 | C11H12N2O2 | [M]+ | Indoles and derivatives | 153-94-6 |
| Indole | 6.31_118.0646 | 4.0317 | C8H7N | [M+H]+ | Indoles and derivatives | 120-72-9 |
| Indoleacetaldehyde | 3.24_142.0652 | 29.8032 | C10H9NO | [M+H-H2O]+ | Indoles and derivatives | 2591-98-2 |
| 5-Hydroxy-L-tryptophan | 0.81_221.0953 | 14.5819 | C11H12N2O3 | [M+H]+ | Indoles and derivatives | 4350-09-8 |
| N-Acetylserotonin | 8.99_199.1696 | 2.1576 | C12H14N2O2 | [M-H2O-H]- | Indoles and derivatives | 1210-83-9 |
| Xanthine | 1.21_153.0407 | 0.1568 | C5H4N4O2 | [M+H]+ | Imidazopyrimidines | 69-89-6 |
| Guanine | 0.90_152.0566 | 0.4998 | C5H5N5O | [M+H]+ | Imidazopyrimidines | 73-40-5 |
| Adenine | 1.49_134.0459 | 9.6982 | C5H5N5 | [M-H]- | Imidazopyrimidines | 73-24-5 |
| Hypoxanthine | 1.23_135.0300 | 8.8869 | C5H4N4O | [M-H]- | Imidazopyrimidines | 68-94-0 |
| 2-Hydroxybutyric acid | 3.29_104.0498 | 24.0270 | C4H8O3 | [M]+ | Hydroxy acids and derivatives | 600-15-7 |
| L-Malic acid | 0.73_133.0126 | 5.4200 | C4H6O5 | [M-H]- | Hydroxy acids and derivatives | 97-67-6 |
| 1-palmitoylglycerophosphocholine | 8.36_496.3371 | 5.3714 | C24H51NO7P | [M]+ | Glycerophospholipids | 17364-16-8 |
| Glycerophosphocholine | 2.09_258.1084 | 5.2365 | C8H21NO6P | [M]+ | Glycerophospholipids | 28319-77-9 |
| LysoPA(16_0_0_0) | 9.94_409.2447 | 21.0143 | C19H39O7P | [M-H]- | Glycerophospholipids | / |
| Silibinin | 5.30_483.1257 | 5.9529 | C25H22O10 | [M+H]+ | Flavonolignans | 22888-70-6 |
| Baicalein | 6.60_271.2732 | 5.7129 | C15H10O5 | [M+H]+ | Flavonoids | 192224-98-9 |
| Cyanidin 3-glucoside | 5.18_449.1065 | 2.4703 | C21H21O11 | [M]+ | Flavonoids | 7084-24-4 |
| Isorhamnetin | 5.02_317.0654 | 0.0762 | C16H12O7 | [M+H]+ | Flavonoids | 480-19-3 |
| 5,7-Dihydroxyflavone | 7.22_253.0503 | 0.0055 | C15H10O4 | [M-H]- | Flavonoids | 480-40-0 |
| Apigenin | 6.23_269.0456 | 5.7129 | C15H10O5 | [M-H]- | Flavonoids | 520-36-5 |
| Kaempferol | 11.47_285.0388 | 5.4339 | C15H10O6 | [M-H]- | Flavonoids | 520-18-3 |
| (-)-Epigallocatechin | 1.20_306.0767 | 8.8213 | C15H14O7 | [M]- | Flavonoids | 970-74-1 |
| Luteolin | 5.84_286.0436 | 5.4339 | C15H10O6 | [M]- | Flavonoids | 491-70-3 |
| Neohesperidin | 5.02_609.1798 | 4.4716 | C28H34O15 | [M-H]- | Flavonoids | 13241-33-3 |
| FMN | 3.43_455.0964 | 2.0303 | C17H21N4O9P | [M-H]- | Flavin nucleotides | 146-17-8 |
| L-2-Hydroxyglutaric acid | 0.56_148.9762 | 5.6084 | C5H8O5 | [M+H]+ | Fatty Acyls | 13095-48-2 |
| Pelargonic acid | 9.65_158.9860 | 2.0631 | C9H18O2 | [M+H]+ | Fatty Acyls | 112-05-0 |
| Pimelic acid | 4.44_160.076 | 14.9929 | C7H12O4 | [M]+ | Fatty Acyls | 111-16-0 |
| Dodecanoic acid | 5.97_200.1646 | 2.9902 | C12H24O2 | [M]+ | Fatty Acyls | 143-07-7 |
| Methyl jasmonate | 9.68_223.9881 | 0.9385 | C13H20O3 | [M]+ | Fatty Acyls | 1211-29-6 |
| Palmitic acid | 10.24_256.2631 | 0.9895 | C16H32O2 | [M]+ | Fatty Acyls | 21096 |
| Gamma-Linolenic acid | 7.79_279.2299 | 7.0057 | C18H30O2 | [M+H]+ | Fatty Acyls | 506-26-3 |
| Linoleic acid | 9.79_280.2632 | 11.1459 | C18H32O2 | [M]+ | Fatty Acyls | 60-33-3 |
| 8,11,14-Eicosatrienoic acid | 8.39_307.2623 | 2.8510 | C20H34O2 | [M+H]+ | Fatty Acyls | 1783-84-2 |
| Arachidic acid | 10.66_312.3258 | 1.9074 | C20H40O2 | [M]+ | Fatty Acyls | 506-30-9 |
| Prostaglandin F2a | 10.24_355.2811 | 3.2431 | C20H34O5 | [M+H]+ | Fatty Acyls | 551-11-1 |
| Erucic acid | 10.19_338.3409 | 0.0878 | C22H42O2 | [M]+ | Fatty Acyls | 112-86-7 |
| Nervonic acid | 9.34_367.3562 | 2.3846 | C24H46O2 | [M+H]+ | Fatty Acyls | 506-37-6 |
| Tetracosanoic acid | 8.88_369.2991 | 8.5040 | C24H48O2 | [M+H]+ | Fatty Acyls | 557-59-5 |
| Myristoleic acid | 7.84_209.1897 | 18.3279 | C14H26O2 | [M+H-H2O]+ | Fatty Acyls | 544-64-9 |
| Stearidonic acid | 10.25_277.2147 | 5.3244 | C18H28O2 | [M+H]+ | Fatty Acyls | 20290-75-9 |
| Oleic acid | 10.41_265.2521 | 13.7002 | C18H34O2 | [M+H-H2O]+ | Fatty Acyls | 112-80-1 |
| 13S-hydroxyoctadecadienoic acid | 9.25_279.2316 | 2.8650 | C18H32O3 | [M+H-H2O]+ | Fatty Acyls | 5204-88-6 |
| 9-OxoODE | 9.99_277.2147 | 9.5017 | C18H30O3 | [M+H-H2O]+ | Fatty Acyls | 54232-59-6 |
| 13-L-Hydroperoxylinoleic acid | 8.86_295.2257 | 10.2770 | C18H32O4 | [M+H-H2O]+ | Fatty Acyls | 33964-75-9 |
| 19(S)-HETE | 9.89_321.2422 | 0.5479 | C20H32O3 | [M+H]+ | Fatty Acyls | 79551-85-2 |
| Prostaglandin D2 | 8.74_335.2197 | 6.3660 | C20H32O5 | [M+H-H2O]+ | Fatty Acyls | 41598-07-6 |
| 4-Acetylbutyrate | 3.22_130.0656 | 19.9899 | C6H10O3 | [M]+ | Fatty Acyls | 3128-06-1 |
| 12,13-DHOME | 8.54_297.2417 | 11.5529 | C18H34O4 | [M+H-H2O]+ | Fatty Acyls | / |
| 2-Oxo-4-methylthiobutanoic acid | 6.12_149.0243 | 3.6535 | C5H8O3S | [M+H]+ | Fatty Acyls | 583-92-6 |
| 5-Acetamidovalerate | 4.43_159.0918 | 14.4571 | C7H13NO3 | [M]+ | Fatty Acyls | / |
| Dethiobiotin | 2.46_215.1392 | 0.1920 | C10H18N2O3 | [M+H]+ | Fatty Acyls | 533-48-2 |
| Prostaglandin B2 | 7.58_335.2198 | 1.3692 | C20H30O4 | [M+H]+ | Fatty Acyls | 13367-85-6 |
| Prostaglandin C1 | 7.79_337.2355 | 0.6529 | C20H32O4 | [M+H]+ | Fatty Acyls | 35687-86-6 |
| Prostaglandin I2 | 7.49_352.2471 | 6.3660 | C20H32O5 | [M]+ | Fatty Acyls | 35121-78-9 |
| Caprylic acid | 1.18_125.0958 | 6.4750 | C8H16O2 | [M-H2O-H]- | Fatty Acyls | 124-07-2 |
| Azelaic acid | 9.85_187.0964 | 0.0103 | C9H16O4 | [M-H]- | Fatty Acyls | 123-99-9 |
| Alpha-Linolenic acid | 11.39_277.2168 | 7.0057 | C18H30O2 | [M-H]- | Fatty Acyls | 463-40-1 |
| Sebacic acid | 7.84_183.1014 | 3.8776 | C10H18O4 | [M-H2O-H]- | Fatty Acyls | 111-20-6 |
| 16-Hydroxy hexadecanoic acid | 8.91_272.2311 | 14.6934 | C16H32O3 | [M]- | Fatty Acyls | 506-13-8 |
| Palmitoleic acid | 10.16_254.2207 | 15.3410 | C16H30O2 | [M]- | Fatty Acyls | 373-49-9 |
| 9,10-Epoxyoctadecenoic acid | 8.25_295.2267 | 2.8650 | C18H32O3 | [M-H]- | Fatty Acyls | / |
| Bovinic acid | 11.39_280.2353 | 11.1459 | C18H32O2 | [M]- | Fatty Acyls | 2540-56-9 |
| Stearic acid | 11.39_284.2672 | 15.1266 | C18H36O2 | [M]- | Fatty Acyls | 57-11-4 |
| 9(S)-HPODE | 9.32_311.2222 | 10.2770 | C18H32O4 | [M-H]- | Fatty Acyls | 29774-12-7 |
| Arachidonic acid | 10.88_303.2310 | 6.3450 | C20H32O2 | [M-H]- | Fatty Acyls | 506-32-1 |
| 5-KETE | 8.89_317.2119 | 0.9457 | C20H30O3 | [M-H]- | Fatty Acyls | 126432-17-5 |
| Pentadecanoic acid | 10.42_241.2169 | 1.6757 | C15H30O2 | [M-H]- | Fatty Acyls | 1002-84-2 |
| EPA (d5) | 10.17_301.2174 | 0.2523 | C20H30O2 | [M-H]- | Fatty Acyls | 10417-94-4 |
| 9,10-DHOME | 8.01_313.2383 | 11.5529 | C18H34O4 | [M-H]- | Fatty Acyls | 263399-34-4 |
| Behenic acid | 10.30_339.3263 | 1.5442 | C22H44O2 | [M-H]- | Fatty Acyls | 112-85-6 |
| (-)-Jasmonic acid | 4.41_209.1180 | 1.5494 | C12H18O3 | [M-H]- | Fatty Acyls | 6894-38-8 |
| Hexadecanedioate | 7.24_286.2105 | 13.6263 | C16H30O4 | [M]- | Fatty Acyls | 505-54-4 |
| 10-Nitrolinoleic acid | 7.97_306.2074 | 1.6002 | C18H31NO4 | [M-H2O-H]- | Fatty Acyls | 774603-04-2 |
| Uracil | 7.10_113.1071 | 2.8458 | C4H4N2O2 | [M+H]+ | Diazines | 66-22-8 |
| Dihydrouracil | 8.54_115.0539 | 29.3526 | C4H6N2O2 | [M+H]+ | Diazines | 504-07-4 |
| Thiopental | 1.55_241.1084 | 28.1035 | C11H18N2O2S | [M-H]- | Diazines | 76-75-5 |
| Aflatoxin G2 | 6.81_330.0699 | 12.2786 | C17H14O7 | [M]- | Coumarins and derivatives | 7241-98-7 |
| 4-Hydroxycinnamic acid | 4.44_165.0548 | 1.2117 | C9H8O3 | [M+H]+ | Cinnamic acids and derivatives | 7400-08-0 |
| trans-Ferulic acid | 8.57_195.1378 | 0.9275 | C10H10O4 | [M+H]+ | Cinnamic acids and derivatives | 537-98-4 |
| Sinapic acid | 6.09_207.0654 | 20.9306 | C11H12O5 | [M+H-H2O]+ | Cinnamic acids and derivatives | 530-59-6 |
| Dattelic acid | 5.45_337.0917 | 0.0610 | C16H16O8 | [M+H]+ | Cinnamic acids and derivatives | 73263-62-4 |
| (10S)-Juvenile hormone III diol phosphate | 7.74_347.1613 | 10.4679 | C16H29O7P | [M+H-H2O]+ | Carboxylic acids and derivatives | / |
| Gulonic acid | 5.72_196.0605 | 11.2212 | C6H12O7 | [M]+ | Carboxylic acids and derivatives | 20246-53-1 |
| 9,10-12,13-Diepoxyoctadecanoate | 7.00_295.2257 | 10.2770 | C18H32O4 | [M+H-H2O]+ | Carboxylic acids and derivatives | / |
| DG(16_0_18_1(9Z)_0_0) | 9.04_577.5232 | 14.4308 | C37H70O5 | [M+H-H2O]+ | Carboxylic acids and derivatives | 3123-73-7 |
| Creatinine | 0.84_114.0665 | 0.2008 | C4H7N3O | [M+H]+ | Carboxylic acids and derivatives | 60-27-5 |
| L-Proline | 0.91_116.0708 | 0.0860 | C5H9NO2 | [M+H]+ | Carboxylic acids and derivatives | 147-85-3 |
| Betaine | 2.45_118.0861 | 1.4904 | C5H11NO2 | [M+H]+ | Carboxylic acids and derivatives | 107-43-7 |
| Pyroglutamic acid | 2.78_130.0495 | 2.8912 | C5H7NO3 | [M+H]+ | Carboxylic acids and derivatives | 98-79-3 |
| Creatine | 0.85_132.0764 | 2.1031 | C4H9N3O2 | [M+H]+ | Carboxylic acids and derivatives | 57-00-1 |
| Glutaric acid | 0.57_131.9737 | 3.6532 | C5H8O4 | [M]+ | Carboxylic acids and derivatives | 110-94-1 |
| cis-4-Hydroxy-D-proline | 1.39_132.0656 | 27.6043 | C5H9NO3 | [M+H]+ | Carboxylic acids and derivatives | 2584-71-6 |
| L-Asparagine | 0.82_133.0608 | 0.1804 | C4H8N2O3 | [M+H]+ | Carboxylic acids and derivatives | 70-47-3 |
| L-Aspartic acid | 0.97_134.0447 | 0.0719 | C4H7NO4 | [M+H]+ | Carboxylic acids and derivatives | 56-84-8 |
| L-Lysine | 0.75_147.1121 | 4.5951 | C6H14N2O2 | [M+H]+ | Carboxylic acids and derivatives | 56-87-1 |
| L-Methionine | 1.60_150.0587 | 2.8256 | C5H11NO2S | [M+H]+ | Carboxylic acids and derivatives | 63-68-3 |
| L-Histidine | 0.76_156.0769 | 0.0602 | C6H9N3O2 | [M+H]+ | Carboxylic acids and derivatives | 71-00-1 |
| 4,5-Dihydroorotic acid | 3.29_158.9605 | 4.4801 | C5H6N2O4 | [M+H]+ | Carboxylic acids and derivatives | 155-54-4 |
| Acetylcysteine | 2.70_163.0414 | 13.0266 | C5H9NO3S | [M]+ | Carboxylic acids and derivatives | 616-91-1 |
| L-Arginine | 0.76_175.1180 | 5.4310 | C6H14N4O2 | [M+H]+ | Carboxylic acids and derivatives | 74-79-3 |
| N-Formyl-L-methionine | 3.14_176.9719 | 0.0378 | C6H11NO3S | [M]+ | Carboxylic acids and derivatives | 4289-98-9 |
| N-Alpha-acetyllysine | 3.23_188.0706 | 0.7106 | C8H16N2O3 | [M]+ | Carboxylic acids and derivatives | 1946-82-3 |
| DL-Homocystine | 10.19_268.2632 | 2.1003 | C8H16N2O4S2 | [M]+ | Carboxylic acids and derivatives | 870-93-9 |
| N6-Acetyl-L-lysine | 0.91_189.1231 | 0.7106 | C8H16N2O3 | [M+H]+ | Carboxylic acids and derivatives | 692-04-6 |
| Ergothioneine | 0.89_230.0954 | 1.7384 | C9H16N3O2S | [M+H]+ | Carboxylic acids and derivatives | 497-30-3 |
| Succinic acid | 1.21_119.0353 | 11.9628 | C4H6O4 | [M+H]+ | Carboxylic acids and derivatives | 110-15-6 |
| N-Acetyl-L-aspartic acid | 0.97_176.0551 | 1.4109 | C6H9NO5 | [M+H]+ | Carboxylic acids and derivatives | 997-55-7 |
| L-Phenylalanine | 1.73_166.0852 | 6.4786 | C9H11NO2 | [M+H]+ | Carboxylic acids and derivatives | 63-91-2 |
| L-2,4-diaminobutyric acid | 7.53_118.0653 | 0.0063 | C4H10N2O2 | [M]+ | Carboxylic acids and derivatives | 1758-80-1 |
| 4-Guanidinobutanoic acid | 3.23_145.0841 | 6.8926 | C5H11N3O2 | [M]+ | Carboxylic acids and derivatives | 463-00-3 |
| (3S,5S)-3,5-Diaminohexanoate | 2.88_146.1175 | 4.5951 | C6H14N2O2 | [M]+ | Carboxylic acids and derivatives | 17027-83-7 |
| N-a-Acetylcitrulline | 5.21_217.1069 | 1.4424 | C8H15N3O4 | [M]+ | Carboxylic acids and derivatives | 33965-42-3 |
| Saccharopine | 0.90 _276.1187 | 0.1334 | C11H20N2O6 | [M]+ | Carboxylic acids and derivatives | 997-68-2 |
| L-Gulonolactone | 0.75_159.0284 | 5.7222 | C6H10O6 | [M-H2O-H]- | Carboxylic acids and derivatives | 1128-23-0 |
| (R)-10-Hydroxystearate | 6.71_281.2452 | 9.9912 | C18H36O3 | [M-H2O-H]- | Carboxylic acids and derivatives | 638-26-6 |
| D-Ornithine | 0.90_131.0812 | 10.8634 | C5H12N2O2 | [M-H]- | Carboxylic acids and derivatives | 348-66-3 |
| D-Glucuronic acid | 1.50_175.0233 | 5.7706 | C6H10O7 | [M-H2O-H]- | Carboxylic acids and derivatives | 1700908 |
| 9,12,13-TriHOME | 7.47_311.2223 | 0.2892 | C18H34O5 | [M-H2O-H]- | Carboxylic acids and derivatives | 97134-11-7 |
| Fumaric acid | 0.75_115.0022 | 2.4309 | C4H4O4 | [M-H]- | Carboxylic acids and derivatives | 110-17-8 |
| 5-Aminopentanoic acid | 0.53_116.9272 | 1.4904 | C5H11NO2 | [M]- | Carboxylic acids and derivatives | 660-88-8 |
| L-Valine | 6.71_116.0699 | 1.4904 | C5H11NO2 | [M-H]- | Carboxylic acids and derivatives | 72-18-4 |
| L-Isoleucine | 6.30_130.0855 | 5.4713 | C6H13NO2 | [M-H]- | Carboxylic acids and derivatives | 73-32-5 |
| L-Glutamine | 0.85_145.0606 | 2.3687 | C5H10N2O3 | [M-H]- | Carboxylic acids and derivatives | 56-85-9 |
| L-Glutamic acid | 0.80_146.0448 | 0.5211 | C5H9NO4 | [M-H]- | Carboxylic acids and derivatives | 56-86-0 |
| L-Tyrosine | 0.96_180.0649 | 4.3030 | C9H11NO3 | [M-H]- | Carboxylic acids and derivatives | 60-18-4 |
| N-Acetylglutamic acid | 3.63_188.1284 | 21.7226 | C7H11NO5 | [M-H]- | Carboxylic acids and derivatives | 1188-37-0 |
| Citric acid | 0.72_191.0188 | 0.2299 | C6H8O7 | [M-H]- | Carboxylic acids and derivatives | 77-92-9 |
| Beta-Leucine | 9.47_130.0868 | 5.4713 | C6H13NO2 | [M-H]- | Carboxylic acids and derivatives | 5699-54-7 |
| N2-gamma-Glutamylglutamine | 0.78_274.1031 | 4.8303 | C10H17N3O6 | [M-H]- | Carboxylic acids and derivatives | 10148-81-9 |
| Palmitoylethanolamide | 9.89_300.2887 | 3.2502 | C18H37NO2 | [M+H]+ | Carboximidic acids and derivatives | 544-31-0 |
| N-Carbamoylputrescine | 6.70_132.1014 | 3.9270 | C5H13N3O | [M+H]+ | Carboximidic acids and derivatives | 6851-51-0 |
| Oxidized glutathione | 0.72_611.1527 | 13.0509 | C20H32N6O12S2 | [M-H]- | Carboximidic acids and derivatives | 27025-41-8 |
| (2R)-2-Hydroxy-3-(phosphonatooxy)propanoate | 0.72_184.9846 | 5.5356 | C3H7O7P | [M-H]- | Carbohydrates and carbohydrate conjugates | 3443-57-0 |
| 3,4-Methylenedioxyamphetamine | 2.97_194.1158 | 0.4121 | C11H15NO2 | [M+H]+ | Benzodioxoles | 4764-17-4 |
| Piperonal | 4.45_151.0388 | 1.1653 | C8H6O3 | [M+H]+ | Benzodioxoles | 120-57-0 |
| Oxazepam | 7.18_285.0386 | 17.6257 | C15H11ClN2O2 | [M-H]- | Benzodiazepines | 604-75-1 |
| beta-Zearalanol | 6.17_305.1751 | 14.8573 | C18H26O5 | [M+H-H2O]+ | Benzene and substituted derivatives | 42422-68-4 |
| Zeranol | 7.43_323.1853 | 14.8573 | C18H26O5 | [M+H]+ | Benzene and substituted derivatives | 26538-44-3 |
| Phenylacetic acid | 8.57_137.1324 | 0.0349 | C8H8O2 | [M+H]+ | Benzene and substituted derivatives | 103-82-2 |
| Phenylacetaldehyde | 2.62_120.0233 | 19.0712 | C8H8O | [M]+ | Benzene and substituted derivatives | 122-78-1 |
| Tyramine | 1.49_138.0913 | 0.5504 | C8H11NO | [M+H]+ | Benzene and substituted derivatives | 51-67-2 |
| 4-Hydroxybenzoic acid | 9.21_121.0285 | 2.1715 | C7H6O3 | [M+H-H2O]+ | Benzene and substituted derivatives | 99-96-7 |
| 4-Hydroxystyrene | 7.50_121.0286 | 19.0712 | C8H8O | [M+H]+ | Benzene and substituted derivatives | 2628-17-3 |
| 2-Hydroxy-6-pentadecylbenzoic acid | 8.90_349.2727 | 2.7944 | C22H36O3 | [M+H]+ | Benzene and substituted derivatives | 16611-84-0 |
| Verapamil | 8.99_455.3028 | 27.0677 | C27H38N2O4 | [M+H]+ | Benzene and substituted derivatives | 52-53-9 |
| Dibutyl phthalate | 9.21_279.1590 | 0.2722 | C16H22O4 | [M+H]+ | Benzene and substituted derivatives | 84-74-2 |
| 2-Phenylethanol | 8.91_105.0703 | 0.0111 | C8H10O | [M+H-H2O]+ | Benzene and substituted derivatives | 22258 |
| Benzaldehyde | 6.89_107.0492 | 0.2242 | C7H6O | [M+H]+ | Benzene and substituted derivatives | 100-52-7 |
| 1,2,3-Trihydroxybenzene | 3.98_127.0399 | 4.1568 | C6H6O3 | [M+H]+ | Benzene and substituted derivatives | 87-66-1 |
| (R)-mandelic Acid | 3.92_135.0441 | 0.0216 | C8H8O3 | [M+H-H2O]+ | Benzene and substituted derivatives | 611-71-2 |
| 4-Hydroxyphenylacetaldehyde | 1.87_136.0621 | 0.0349 | C8H8O2 | [M]+ | Benzene and substituted derivatives | 7339-87-9 |
| p-Aminobenzoic acid | 1.15_138.0543 | 3.2490 | C7H7NO2 | [M+H]+ | Benzene and substituted derivatives | 150-13-0 |
| 2-Pyrocatechuic acid | 4.47_154.0427 | 6.0807 | C7H6O4 | [M]+ | Benzene and substituted derivatives | 303-38-8 |
| Diphenylamine | 11.42_170.0952 | 4.0254 | C12H11N | [M+H]+ | Benzene and substituted derivatives | 122-39-4 |
| Methylisoeugenol | 7.64_179.1067 | 0.1340 | C11H14O2 | [M+H]+ | Benzene and substituted derivatives | 6379-72-2 |
| Dulcin | 1.20_181.0967 | 1.8574 | C9H12N2O2 | [M+H]+ | Benzene and substituted derivatives | 150-69-6 |
| Cyclizine | 6.28_266.1719 | 9.0853 | C18H22N2 | [M]+ | Benzene and substituted derivatives | 82-92-8 |
| Dihydrocapsaicin | 5.65_308.2212 | 1.7963 | C18H29NO3 | [M+H]+ | Benzene and substituted derivatives | 19408-84-5 |
| Phenylethylamine | 4.60_121.0287 | 2.7395 | C8H11N | [M]- | Benzene and substituted derivatives | 64-04-0 |
| Homogentisic acid | 11.37_149.0085 | 2.4906 | C8H8O4 | [M-H2O-H]- | Benzene and substituted derivatives | 451-13-8 |
| Phthalic acid | 9.14_165.0396 | 2.6603 | C8H6O4 | [M-H]- | Benzene and substituted derivatives | 88-99-3 |
| Labetalol | 8.00_328.1840 | 16.1495 | C19H24N2O3 | [M]- | Benzene and substituted derivatives | 36894-69-6 |
| 2-Ethylhexyl phthalate | 6.49_277.1450 | 0.2722 | C16H22O4 | [M-H]- | Benzene and substituted derivatives | 4376-20-9 |
| 5-(2-Hydroxyethyl)-4-methylthiazole | 5.24_143.0396 | 0.2395 | C6H9NOS | [M]+ | Azoles | 137-00-8 |
| 1-Methylhistidine | 0.73_170.0941 | 10.1357 | C7H11N3O2 | [M+H]+ | Azacyclic compounds | 332-80-9 |
| GDP | 3.59_444.0410 | 21.2237 | C10H15N5O11P2 | [M+H]+ | Azacyclic compounds | 146-91-8 |
| CTP | 0.69_481.9792 | 4.0998 | C9H16N3O14P3 | [M-H]- | Azacyclic compounds | 65-47-4 |
| dGTP | 0.71_505.9839 | 8.9409 | C10H16N5O13P3 | [M-H]- | Azacyclic compounds | 2564-35-4 |
| Bovinocidin | 9.71_120.0256 | 29.4407 | C3H5NO4 | [M+H]+ | Allyl-type 1,3-dipolar organic compounds | 504-88-1 |
| Pantothenic acid | 1.86_220.1180 | 0.1090 | C9H17NO5 | [M+H]+ | Alcohols and polyols | 79-83-4 |
| Isochlorogenic acid b | 5.57_499.1242 | 9.6850 | C25H24O12 | [M+H-H2O]+ | Alcohols and polyols | 14534-61-3 |
| (-)-Bornesitol | 8.66_194.0809 | 1.5802 | C7H14O6 | [M]+ | Alcohols and polyols | 484-71-9 |
| 5'-Methylthioadenosine | 0.73_298.0957 | 3.9450 | C11H15N5O3S | [M+H]+ | 5'-deoxyribonucleosides | 2457-80-9 |
| S-Adenosylhomocysteine | 11.00_384.3455 | 4.5513 | C14H20N6O5S | [M]+ | 5'-deoxyribonucleosides | 979-92-0 |
| NAD | 0.86_664.1083 | 12.1968 | C21H28N7O14P2 | [M+H]+ | (5'->5')-dinucleotides | 53-84-9 |
| NADH | 1.17_664.1175 | 0.0000 | C21H29N7O14P2 | [M-H]- | (5'->5')-dinucleotides | 58-68-4 |
| trans-Cinnamate | 0.85_149.0639 | 28.3368 | C9H8O2 | [M+H]+ | unclassified | 140-10-3 |
| 3-Amino-4-hydroxybenzoate | 1.07_153.998 | 2.3709 | C7H7NO3 | [M+H]+ | unclassified | 1571-72-8 |
| Anabasine | 6.54_163.0399 | 3.1714 | C10H14N2 | [M+H]+ | unclassified | 13078-04-1 |
| L-4-Hydroxyphenylglycine | 0.70_167.0127 | 1.0646 | C8H9NO3 | [M]+ | unclassified | 32462-30-9 |
| 10-Hydroxydecanoic acid | 8.92_171.1378 | 23.5716 | C10H20O3 | [M+H-H2O]+ | unclassified | 1679-53-4 |
| 1-Hydroxy-2-naphthoate | 5.71_171.1483 | 3.3889 | C11H8O3 | [M+H-H2O]+ | unclassified | 86-48-6 |
| 3-Dehydroshikimate | 0.58_171.9915 | 8.8907 | C7H8O5 | [M]+ | unclassified | 2922-42-1 |
| Lumichrome | 5.30_243.0871 | 2.3695 | C12H10N4O2 | [M+H]+ | unclassified | 1086-80-2 |
| Fisetin | 6.31_287.0547 | 2.0066 | C15H10O6 | [M+H]+ | unclassified | 345909-34-4 |
| Glutathione | 0.93_307.0821 | 1.6511 | C10H17N3O6S | [M]+ | unclassified | 70-18-8 |
| S-Hexyl-glutathione | 9.49_391.2837 | 0.0043 | C16H29N3O6S | [M]+ | unclassified | 24425-56-7 |
| Sodium deoxycholate | 9.52_414.3214 | 2.8621 | C24H39O4. Na | [M]+ | unclassified | 302-95-4 |
| Deoxyuridine-5'-triphosphate | 9.86_468.3903 | 0.3242 | C9H15N2O14P3 | [M+H]+ | unclassified | 102814-08-4 |
| beta-Carotene | 10.28_536.1648 | 0.8259 | C40H56 | [M]+ | unclassified | 7235-40-7 |
| Rutin | 11.11_610.1806 | 6.6068 | C27H30O16 | [M]+ | unclassified | 207671-50-9 |
| 9(S)-HPOT | 8.50_293.2119 | 1.8784 | C18H30O4 | [M+H-H2O]+ | unclassified | 111004-08-1 |
| Catechol | 11.37_111.0203 | 4.5520 | C6H6O2 | [M+H]+ | unclassified | 120-80-9 |
| 7-Methylcapillarisin | 7.43_315.0856 | 2.1454 | C17H14O6 | [M+H]+ | unclassified | 6601-62-3 |
| (4Z,7Z,10Z,13Z,16Z,19Z)-Docosahexaenoic acid ethyl ester | 7.22_357.2787 | 0.2127 | C24H36O2 | [M+H]+ | unclassified | 81926-94-5 |
| Lupeol | 11.14_409.3814 | 6.4341 | C30H50O | [M+H-H2O]+ | unclassified | 545-47-1 |
| (R)-5,6-Dihydrothymine | 1.93_128.0701 | 4.0040 | C5H8N2O2 | [M]+ | unclassified | 86387-01-1 |
| 4-Oxoproline | 7.32_130.0495 | 2.8912 | C5H7NO3 | [M+H]+ | unclassified | 4347-18-6 |
| (S)-4-Amino-5-oxopentanoate | 3.24_132.0808 | 27.6043 | C5H9NO3 | [M+H]+ | unclassified | 68462-55-5 |
| threo-3-Hydroxy-D-aspartate | 10.07_149.0226 | 4.5899 | C4H7NO5 | [M]+ | unclassified | / |
| erythro-3-Hydroxy-Ls-aspartate | 2.60_149.0243 | 4.5899 | C4H7NO5 | [M]+ | unclassified | / |
| (-)-Isopiperitenone | 7.60_151.1114 | 1.7409 | C10H14O | [M+H]+ | unclassified | 80995-97-7 |
| 4-Hydroxycoumarin | 5.57_163.0398 | 3.5101 | C9H6O3 | [M+H]+ | unclassified | 1076-38-6 |
| Isosafrole | 8.97 _163.0753 | 0.4660 | C10H10O2 | [M+H]+ | unclassified | 120-58-1 |
| 2-Deoxystreptamine | 6.90_163.1117 | 0.0037 | C6H14N2O3 | [M+H]+ | unclassified | 2037-48-1 |
| 8-Amino-7-oxononanoate | 4.58_188.1283 | 0.0573 | C9H17NO3 | [M+H]+ | unclassified | 4707-58-8 |
| N-Butyryl-L-homoserine lactone | 3.89_172.0968 | 0.1395 | C8H13NO3 | [M+H]+ | unclassified | 67605-85-0 |
| 2-Hydroxy-3-oxoadipate | 4.62_177.0547 | 0.0316 | C6H8O6 | [M+H]+ | unclassified | / |
| L-Homophenylalanine | 3.86_180.1029 | 2.7289 | C10H13NO2 | [M+H]+ | unclassified | 943-73-7 |
| 5-Guanidino-3-methyl-2-oxopentanoate | 0.93_187.1079 | 0.5028 | C7H13N3O3 | [M]+ | unclassified | / |
| 3-Methyl-L-tyrosine | 8.11_195.1015 | 0.0405 | C10H13NO3 | [M]+ | unclassified | / |
| (1S,2R,4S)-(-)-Bornyl acetate | 8.92_197.1535 | 0.0406 | C12H20O2 | [M+H]+ | unclassified | 5655-61-8 |
| D-Lysopine | 0.87_219.1334 | 1.8451 | C9H18N2O4 | [M+H]+ | unclassified | 34522-31-1 |
| (2S)-Flavanone | 6.52_224.0918 | 0.3115 | C15H12O2 | [M]+ | unclassified | 487-26-3 |
| Butyryl-L-carnitine | 2.19_232.1542 | 0.0034 | C11H21NO4 | [M+H]+ | unclassified | 25576-40-3 |
| Xanthoxin | 7.49_233.1535 | 17.3019 | C15H22O3 | [M+H-H2O]+ | unclassified | 8066-07-7 |
| Lenacil | 2.32_235.1436 | 1.5960 | C13H18N2O2 | [M+H]+ | unclassified | 2164-08-1 |
| Confertifolin | 8.28 _235.1688 | 0.4746 | C15H22O2 | [M+H]+ | unclassified | 1811-23-0 |
| N-Acetyl-D-tryptophan | 2.98_247.1290 | 0.8631 | C13H14N2O3 | [M+H]+ | unclassified | 2280-01-5 |
| Juvenile hormone III | 8.98_249.1847 | 15.7875 | C16H26O3 | [M+H-H2O]+ | unclassified | 22963-93-5 |
| (10S)-Juvenile hormone III diol | 7.47_267.1955 | 15.4718 | C16H28O4 | [M+H-H2O]+ | unclassified | / |
| Neocembrene | 9.30_273.2569 | 1.2319 | C20H32 | [M+H]+ | unclassified | 31570-39-5 |
| 3-Ketosphingosine | 9.24_297.2413 | 3.5804 | C18H35NO2 | [M]+ | unclassified | / |
| 13(S)-HpOTrE | 8.01_293.2116 | 15.7721 | C18H30O4 | [M+H-H2O]+ | unclassified | 67597-26-6 |
| Methoprene | 9.70_293.2470 | 0.1788 | C19H34O3 | [M+H-H2O]+ | unclassified | 40596-69-8 |
| Nopaline | 1.45 _304.1501 | 0.2925 | C11H20N4O6 | [M]+ | unclassified | 22350-70-5 |
| 5(S)-HpETE | 8.37_319.2260 | 1.8637 | C20H32O4 | [M+H-H2O]+ | unclassified | 71774-08-8 |
| 12(S)-HpETE | 7.56 _319.2259 | 2.5793 | C20H32O4 | [M+H-H2O]+ | unclassified | 71774-10-2 |
| Misoprostol | 8.43_369.2623 | 3.3858 | C21H36O5 | [M+H]+ | unclassified | 59122-46-2 |
| Ergosta-5,7,22,24(28)-tetraen-3beta-ol | 10.23_377.3259 | 25.7973 | C28H42O | [M+H-H2O]+ | unclassified | 29560-24-5 |
| 1-Arachidonoylglycerol | 9.10 _379.2835 | 2.0460 | C23H38O4 | [M+H]+ | unclassified | 124511-15-5 |
| 5alpha-Ergosta-7,22-diene-3beta,5-diol | 10.54_415.3555 | 3.7943 | C28H46O2 | [M+H]+ | unclassified | / |
| 3-Dehydroecdysone | 7.54_445.2945 | 8.6100 | C27H42O6 | [M+H-H2O]+ | unclassified | 39750-00-0 |
| Antibiotic JI-20A | 9.47_482.3206 | 7.4917 | C19H39N5O9 | [M+H]+ | unclassified | 51846-97-0 |
| Decarbamoylnovobiocin | 8.61_570.2765 | 12.3910 | C30H35NO10 | [M+H]+ | unclassified | 10544-02-2 |
| Phenyl acetate | 9.43_134.8933 | 0.0349 | C8H8O2 | [M-H]- | unclassified | 122-79-2 |
| Dehydroascorbate | 0.96_173.0079 | 0.0234 | C6H6O6 | [M-H]- | unclassified | 490-83-5 |
| Caffeate | 4.28 _179.0352 | 0.9830 | C9H8O4 | [M-H]- | unclassified | 501-16-6 |
| Ascorbate | 0.78_175.0238 | 0.0316 | C6H8O6 | [M-H]- | unclassified | 50-81-7 |
| Quinate | 1.25_191.0554 | 0.0555 | C7H12O6 | [M-H]- | unclassified | 77-95-2 |
| Methyl beta-D-galactoside | 7.63_194.0813 | 1.5802 | C7H14O6 | [M]- | unclassified | 1824-94-8 |
| 3-Oxalomalate | 6.53 _205.0119 | 5.5168 | C6H6O8 | [M-H]- | unclassified | 3687-15-8 |
| 2'-Deoxyadenosine | 9.27_250.1443 | 1.6333 | C10H13N5O3 | [M-H]- | unclassified | 16373-93-6 |
| Taxifolin | 6.70_285.0402 | 1.0174 | C15H12O7 | [M-H2O-H]- | unclassified | 480-18-2 |
| Cellobiose | 0.93 _341.1078 | 0.5598 | C12H22O11 | [M-H]- | unclassified | 16462-44-5 |
| S-Adenosylmethionine | 8.05 _397.2257 | 2.3277 | C15H22N6O5S | [M-H]- | unclassified | 17176-17-9 |
| Guanosine-5'-triphosphate | 0.70_521.9800 | 6.6072 | C10H16N5O14P3 | [M-H]- | unclassified | 36051-31-7 |
| Procollagen 5-hydroxy-L-lysine | 6.53_197.8076 | 2.2468 | C7H13N3O3R2 | [M-H]- | unclassified | 13204-98-3 |
| 3-O-Methylquercetin | 5.85_315.0510 | 0.0762 | C16H12O7 | [M-H]- | unclassified | 1486-70-0 |
| Neochlorogenic acid | 1.93_353.0875 | 0.9176 | C16H18O9 | [M-H]- | unclassified | 906-33-2 |
| Gardenoside | 1.94_403.1238 | 2.0440 | C17H24O11 | [M-H]- | unclassified | 24512-62-7 |

**Table S5.** Result of DEGs by RNA-seq

| ID | Symbol | baseMean | log_2_FC | *p* | FDR | Description |
| --- | --- | --- | --- | --- | --- | --- |
| E3.1.4.46, glpQ, ugpQ | [EC:3.1.4.46] | 16.1611 | 1.8241 | 0.003625 | 0.006671 | K01126 |
| gpmB | [EC:5.4.2.12] | 207.9259 | 1.0525 | 0.000105 | 0.000248 | K15634 |
| K00001 | E1.1.1.1, adh | 6131.8622 | -1.3250 | 1.02E-11 | 4.71E-11 | zinc-dependent alcohol dehydrogenase family protein |
| K00003 | E1.1.1.3 | 1626.0061 | 1.1484 | 6.79E-09 | 2.49E-08 | homoserine dehydrogenase |
| K00005 | gldA | 18065.4892 | 1.1913 | 0.000372 | 0.000803 | glycerol dehydrogenase |
| K00009 | mtlD | 658.6029 | -1.1738 | 1.20E-17 | 9.09E-17 | mannitol-1-phosphate 5-dehydrogenase |
| K00013 | hisD | 920.4955 | 1.9961 | 4.21E-33 | 7.85E-32 | histidinol dehydrogenase |
| K00016 | LDH, ldh | 23777.7905 | 1.6166 | 0.000174 | 0.000395 | L-lactate dehydrogenase |
| K00027 | ME2, sfcA, maeA | 608.9314 | -1.0416 | 0.000195 | 0.000438 | type II toxin-antitoxin system HicA family toxin |
| K00052 | leuB, IMDH | 2893.0859 | 1.3523 | 6.42E-11 | 2.78E-10 | 3-isopropylmalate dehydrogenase |
| K00054 | mvaA | 158.5798 | -1.3045 | 6.56E-06 | 1.82E-05 | hydroxymethylglutaryl-CoA reductase, degradative |
| K00075 | murB | 921.6166 | -1.0476 | 6.74E-08 | 2.27E-07 | UDP-N-acetylmuramate dehydrogenase |
| K00077 | panE, apbA | 56.7158 | -2.1199 | 2.59E-10 | 1.07E-09 | 2-dehydropantoate 2-reductase |
| K00133 | asd | 3221.2207 | 1.2112 | 5.00E-07 | 1.56E-06 | aspartate-semialdehyde dehydrogenase |
| K00134 | GAPDH, gapA | 111837.8805 | 1.5454 | 3.02E-05 | 7.64E-05 | type I glyceraldehyde-3-phosphate dehydrogenase |
| K00249 | ACADM, acd | 264.3977 | -3.5542 | 1.25E-28 | 1.76E-27 | acyl-CoA/acyl-ACP dehydrogenase |
| K00265 | gltB | 7298.9592 | 2.2313 | 2.73E-47 | 1.07E-45 | glutamate synthase large subunit |
| K00266 | gltD | 1751.6140 | 1.6836 | 3.09E-29 | 4.65E-28 | glutamate synthase subunit beta |
| K00287 | DHFR, folA | 131.2486 | -1.0358 | 0.02466 | 0.038714 | dihydrofolate reductase |
| K00355 | NQO1 | 370.9262 | 2.1940 | 3.49E-22 | 3.41E-21 | NAD(P)H-dependent oxidoreductase |
| K00382 | DLD, lpd, pdhD | 1432.1283 | 3.8700 | 1.51E-10 | 6.31E-10 | dihydrolipoyl dehydrogenase |
| K00383 | GSR, gor | 6831.0866 | 4.5123 | 1.29E-73 | 1.37E-71 | NAD(P)/FAD-dependent oxidoreductase |
| K00384 | trxB, TRR | 6936.2309 | -1.7238 | 3.05E-14 | 1.76E-13 | thioredoxin-disulfide reductase |
| K00459 | ncd2, npd | 100.6604 | -1.1065 | 0.000477 | 0.001011 | MarR family transcriptional regulator |
| K00525 | E1.17.4.1A, nrdA, nrdE | 665.9131 | -1.0013 | 0.011275 | 0.018917 | class 1b ribonucleoside-diphosphate reductase subunit alpha |
| K00560 | thyA, TYMS | 2183.5586 | -2.5073 | 5.65E-21 | 5.13E-20 | thymidylate synthase |
| K00600 | glyA, SHMT | 675.4450 | 1.6411 | 3.80E-14 | 2.17E-13 | serine hydroxymethyltransferase |
| K00602 | purH | 791.8381 | 2.2595 | 1.31E-27 | 1.75E-26 | bifunctional phosphoribosylaminoimidazolecarboxamide formyltransferase/IMP cyclohydrolase |
| K00609 | pyrB, PYR2 | 1539.8801 | 3.4127 | 5.31E-128 | 1.20E-125 | aspartate carbamoyltransferase catalytic subunit |
| K00615 | E2.2.1.1, tktA, tktB | 3027.1802 | 1.0741 | 6.92E-06 | 1.91E-05 | transketolase |
| K00616 | E2.2.1.2, talA, talB | 3847.7365 | 1.0201 | 0.019687 | 0.031565 | fructose-6-phosphate aldolase |
| K00625 | E2.3.1.8, pta | 5566.3766 | 1.0945 | 0.002018 | 0.003878 | phosphate acetyltransferase |
| K00627 | DLAT, aceF, pdhC | 307.9220 | 3.7059 | 1.08E-11 | 4.97E-11 | 2-oxo acid dehydrogenase subunit E2 |
| K00645 | fabD | 744.1566 | -2.2545 | 2.61E-11 | 1.18E-10 | mutanobactin A biosynthesis transacylase MubG |
| K00648 | fabH | 872.7840 | -1.5878 | 3.34E-09 | 1.26E-08 | ketoacyl-ACP synthase III |
| K00656 | E2.3.1.54, pflD | 69352.3363 | 2.7218 | 1.97E-13 | 1.06E-12 | formate C-acetyltransferase |
| K00674 | dapD | 961.1139 | 1.2765 | 3.25E-08 | 1.13E-07 | 2,3,4,5-tetrahydropyridine-2,6-dicarboxylate N-acetyltransferase |
| K00689 | E2.4.1.5 | 5237.7401 | -3.3985 | 1.39E-108 | 2.28E-106 | glucosyltransferase-S |
| K00689 | E2.4.1.5 | 3896.1737 | -1.9676 | 2.50E-61 | 1.74E-59 | YSIRK-type signal peptide-containing protein |
| K00690 | E2.4.1.7 | 1075.0275 | 2.1548 | 1.29E-45 | 4.41E-44 | sucrose phosphorylase |
| K00692 | sacB | 7055.5603 | -1.9636 | 3.08E-15 | 1.95E-14 | levansucrase |
| K00759 | APRT, apt | 207.8303 | -1.4302 | 8.06E-07 | 2.47E-06 | adenine phosphoribosyltransferase |
| K00760 | hprT, hpt, HPRT1 | 5955.0212 | -1.8029 | 9.49E-31 | 1.59E-29 | hypoxanthine phosphoribosyltransferase |
| K00761 | upp, UPRT | 4086.2416 | -1.1093 | 4.22E-11 | 1.88E-10 | uracil phosphoribosyltransferase |
| K00763 | pncB, NAPRT1 | 1602.6088 | 1.5173 | 5.37E-11 | 2.33E-10 | nicotinate phosphoribosyltransferase |
| K00764 | purF, PPAT | 1301.2892 | 2.1732 | 1.08E-26 | 1.36E-25 | amidophosphoribosyltransferase |
| K00765 | hisG | 307.0473 | 2.4162 | 4.01E-39 | 1.04E-37 | ATP phosphoribosyltransferase |
| K00766 | trpD | 960.8076 | 1.6783 | 9.71E-15 | 5.91E-14 | anthranilate phosphoribosyltransferase |
| K00790 | murA | 1924.4450 | -1.4654 | 7.51E-20 | 6.46E-19 | UDP-N-acetylglucosamine 1-carboxyvinyltransferase |
| K00799 | GST, gst | 249.0034 | -1.3701 | 2.69E-09 | 1.02E-08 | CYTH domain-containing protein |
| K00806 | uppS | 818.4765 | -2.9574 | 2.72E-28 | 3.70E-27 | isoprenyl transferase |
| K00817 | hisC | 230.9207 | 1.8895 | 7.31E-15 | 4.46E-14 | histidinol-phosphate transaminase |
| K00820 | glmS, GFPT | 3274.1483 | -1.3235 | 2.67E-40 | 7.00E-39 | glutamine--fructose-6-phosphate transaminase (isomerizing) |
| K00831 | serC, PSAT1 | 1283.9921 | 1.0721 | 6.58E-09 | 2.43E-08 | 3-phosphoserine/phosphohydroxythreonine transaminase |
| K00850 | pfkA, PFK | 4320.2136 | 1.0596 | 1.31E-13 | 7.24E-13 | 6-phosphofructokinase |
| K00859 | coaE | 189.3134 | 1.1751 | 3.82E-07 | 1.21E-06 | dephospho-CoA kinase |
| K00867 | coaA | 1709.8785 | -1.1728 | 0.000191 | 0.000429 | type I pantothenate kinase |
| K00868 | pdxK, pdxY | 1616.5368 | 2.4367 | 1.76E-13 | 9.60E-13 | bifunctional hydroxymethylpyrimidine kinase/phosphomethylpyrimidine kinase |
| K00872 | thrB1 | 556.8183 | 1.4445 | 4.58E-22 | 4.35E-21 | homoserine kinase |
| K00882 | fruK | 2697.1290 | 1.3892 | 1.94E-13 | 1.05E-12 | 1-phosphofructokinase |
| K00917 | lacC | 1672.5204 | 1.9285 | 0.000484 | 0.001024 | tagatose-6-phosphate kinase |
| K00919 | ispE | 956.3918 | -1.3053 | 0.000451 | 0.000961 | 4-(cytidine 5'-diphospho)-2-C-methyl-D-erythritol kinase |
| K00928 | lysC | 1245.8115 | -1.2674 | 0.000243 | 0.000535 | aspartate kinase |
| K00938 | E2.7.4.2, mvaK2 | 876.3190 | -1.0856 | 1.14E-05 | 3.06E-05 | phosphomevalonate kinase |
| K00943 | tmk, DTYMK | 731.2778 | -1.6104 | 8.53E-09 | 3.08E-08 | dTMP kinase |
| K00948 | PRPS, prsA | 1489.9052 | -1.9553 | 2.85E-11 | 1.28E-10 | ribose-phosphate diphosphokinase |
| K00965 | galT, GALT | 643.8924 | -1.6578 | 7.56E-09 | 2.77E-08 | cystathionine beta-lyase |
| K00981 | E2.7.7.41, CDS1, CDS2, cdsA | 729.8140 | -1.8898 | 2.43E-11 | 1.10E-10 | phosphatidate cytidylyltransferase |
| K00997 | acpS | 1188.4458 | 2.1867 | 7.68E-51 | 3.47E-49 | holo-ACP synthase |
| K01005 | - | 1326.9372 | -1.4584 | 2.17E-14 | 1.27E-13 | LCP family protein |
| K01079 | serB, PSPH | 327.2011 | 2.1098 | 2.76E-18 | 2.16E-17 | phosphoserine phosphatase SerB |
| K01096 | pgpB | 347.7282 | -1.7990 | 3.65E-23 | 3.75E-22 | phosphatase PAP2 family protein |
| K01176 | AMY, amyA, malS | 1920.0491 | 2.5612 | 2.90E-37 | 6.62E-36 | alpha-amylase |
| K01198 | xynB | 319.8916 | -1.2402 | 3.24E-08 | 1.13E-07 | AraC family transcriptional regulator |
| K01212 | sacC, levB | 403.4088 | -2.4738 | 6.45E-36 | 1.32E-34 | glycoside hydrolase family 32 protein |
| K01220 | E3.2.1.85, lacG | 348.9035 | 1.7625 | 8.24E-09 | 2.99E-08 | 6-phospho-beta-galactosidase |
| K01223 | E3.2.1.86B, bglA | 1833.4690 | 2.3517 | 9.04E-17 | 6.45E-16 | glycoside hydrolase family 1 protein |
| K01243 | mtnN, mtn, pfs | 787.0003 | -1.2146 | 1.45E-09 | 5.59E-09 | 5'-methylthioadenosine/adenosylhomocysteine nucleosidase |
| K01426 | E3.5.1.4, amiE | 135.5453 | 1.2352 | 0.000164 | 0.000374 | DUF1697 domain-containing protein |
| K01443 | nagA, AMDHD2 | 10218.4686 | 1.4369 | 0.000192 | 0.000432 | N-acetylglucosamine-6-phosphate deacetylase |
| K01448 | amiABC | 3251.8809 | -1.5293 | 2.90E-11 | 1.30E-10 | autolysin |
| K01465 | URA4, pyrC | 1734.6690 | 1.5406 | 1.80E-37 | 4.27E-36 | dihydroorotase |
| K01467 | ampC | 4790.8946 | -1.2522 | 1.06E-13 | 5.89E-13 | serine hydrolase |
| K01496 | hisI | 99.1485 | 2.1820 | 8.27E-18 | 6.28E-17 | phosphoribosyl-AMP cyclohydrolase |
| K01515 | nudF | 581.0774 | -1.1179 | 9.37E-10 | 3.70E-09 | NUDIX hydrolase |
| K01519 | ITPA | 2626.9350 | -1.3555 | 1.71E-14 | 1.01E-13 | nucleoside-triphosphate diphosphatase |
| K01523 | hisE | 56.6085 | 2.1134 | 4.64E-11 | 2.05E-10 | phosphoribosyl-ATP diphosphatase |
| K01569 | oxdD | 3544.8104 | 4.0929 | 6.68E-65 | 5.48E-63 | oxalate decarboxylase family bicupin |
| K01571 | oadA | 268.0982 | 1.7672 | 1.27E-15 | 8.35E-15 | oxaloacetate decarboxylase subunit alpha |
| K01588 | purE | 96.6571 | 1.1312 | 0.000156 | 0.000357 | 5-(carboxyamino)imidazole ribonucleotide mutase |
| K01589 | purK | 1864.3026 | 1.0826 | 8.55E-10 | 3.40E-09 | 5-(carboxyamino)imidazole ribonucleotide synthase |
| K01595 | ppc | 18683.7597 | -1.0503 | 2.80E-13 | 1.50E-12 | phosphoenolpyruvate carboxylase |
| K01597 | MVD, mvaD | 888.9889 | -2.0405 | 5.01E-11 | 2.19E-10 | diphosphomevalonate decarboxylase |
| K01598 | PPCDC, coaC | 198.2668 | -2.7768 | 1.31E-20 | 1.15E-19 | phosphopantothenoylcysteine decarboxylase |
| K01599 | hemE, UROD | 244.1701 | -2.2040 | 2.42E-07 | 7.81E-07 | uroporphyrinogen decarboxylase |
| K01607 | pcaC | 32.3179 | 1.6767 | 0.000639 | 0.001333 | carboxymuconolactone decarboxylase family protein |
| K01609 | trpC | 363.3700 | 1.5626 | 2.77E-24 | 3.05E-23 | indole-3-glycerol phosphate synthase TrpC |
| K01635 | lacD | 81.0602 | 1.5684 | 3.76E-06 | 1.07E-05 | tagatose-bisphosphate aldolase |
| K01635 | lacD | 460.1631 | 1.7434 | 0.001724 | 0.003346 | tagatose-bisphosphate aldolase |
| K01641 | E2.3.3.10 | 125.7111 | -1.2364 | 2.33E-06 | 6.74E-06 | hydroxymethylglutaryl-CoA synthase |
| K01643 | citF | 501.1028 | 1.7289 | 7.27E-12 | 3.41E-11 | citrate lyase subunit alpha |
| K01644 | citE | 296.5845 | 1.4484 | 3.64E-07 | 1.16E-06 | citrate (pro-3S)-lyase subunit beta |
| K01646 | citD | 16.6138 | 1.4330 | 0.002487 | 0.004691 | citrate lyase acyl carrier protein |
| K01649 | leuA, IMS | 6759.2710 | 2.2424 | 7.58E-14 | 4.23E-13 | 2-isopropylmalate synthase |
| K01657 | trpE | 150.9730 | 1.1564 | 1.08E-08 | 3.86E-08 | anthranilate synthase component I |
| K01658 | trpG | 97.5845 | 1.0410 | 1.53E-05 | 4.02E-05 | aminodeoxychorismate/anthranilate synthase component II |
| K01674 | cah | 260.6223 | -2.6324 | 1.44E-23 | 1.50E-22 | carbonic anhydrase family protein |
| K01687 | ilvD | 3141.3632 | 1.4577 | 3.30E-11 | 1.48E-10 | dihydroxy-acid dehydratase |
| K01693 | hisB | 308.7027 | 2.1505 | 1.47E-29 | 2.32E-28 | imidazoleglycerol-phosphate dehydratase HisB |
| K01695 | trpA | 1122.4608 | 2.2416 | 1.95E-52 | 1.04E-50 | tryptophan synthase subunit alpha |
| K01696 | trpB | 521.7489 | 2.1594 | 2.43E-29 | 3.76E-28 | tryptophan synthase subunit beta |
| K01703 | leuC, IPMI-L | 3088.5313 | 1.1371 | 0.000272 | 0.000595 | 3-isopropylmalate dehydratase large subunit |
| K01714 | dapA | 1062.1359 | 1.4597 | 2.31E-14 | 1.34E-13 | 4-hydroxy-tetrahydrodipicolinate synthase |
| K01739 | metB | 109.5221 | -1.5515 | 1.69E-09 | 6.53E-09 | cystathionine gamma-synthase |
| K01755 | argH, ASL | 210.7128 | 1.8414 | 7.08E-11 | 3.05E-10 | argininosuccinate lyase |
| K01759 | GLO1, gloA | 1266.3214 | 1.0137 | 0.002345 | 0.004461 | lactoylglutathione lyase |
| K01775 | alr | 5047.9994 | 2.2176 | 2.35E-33 | 4.47E-32 | alanine racemase |
| K01776 | murI | 2253.3342 | -1.4673 | 3.62E-21 | 3.32E-20 | glutamate racemase |
| K01785 | galM, GALM | 549.9042 | -1.8183 | 1.34E-23 | 1.41E-22 | DUF1912 family protein |
| K01808 | rpiB | 3465.4561 | -2.1683 | 2.93E-41 | 8.15E-40 | RpiB/LacA/LacB family sugar-phosphate isomerase |
| K01814 | hisA | 733.3200 | 2.3332 | 4.00E-27 | 5.12E-26 | 1-(5-phosphoribosyl)-5-[(5-phosphoribosylamino)methylideneamino]imidazole-4-carboxamide isomerase |
| K01817 | trpF | 437.8020 | 1.6498 | 2.50E-16 | 1.74E-15 | phosphoribosylanthranilate isomerase |
| K01819 | E5.3.1.26, lacA, lacB | 2132.0790 | 1.5299 | 0.00586 | 0.010341 | galactose-6-phosphate isomerase subunit LacB |
| K01819 | E5.3.1.26, lacA, lacB | 190.0619 | 1.2973 | 0.018099 | 0.029292 | galactose-6-phosphate isomerase subunit LacA |
| K01834 | PGAM, gpmA | 529.1715 | -1.1732 | 1.57E-13 | 8.57E-13 | phosphoglycerate mutase |
| K01835 | pgm | 4656.0700 | 1.6030 | 8.29E-09 | 3.01E-08 | phospho-sugar mutase |
| K01839 | deoB | 1158.5810 | 1.1518 | 3.72E-09 | 1.40E-08 | phosphopentomutase |
| K01873 | VARS, valS | 8682.4844 | -2.4244 | 4.05E-38 | 1.00E-36 | valine--tRNA ligase |
| K01874 | MARS, metG | 1664.7835 | 1.1827 | 1.15E-12 | 5.74E-12 | methionine--tRNA ligase |
| K01875 | SARS, serS | 1678.3890 | 1.0593 | 1.75E-15 | 1.13E-14 | serine--tRNA ligase |
| K01878 | glyQ | 594.6227 | 2.2649 | 5.69E-28 | 7.61E-27 | glycine--tRNA ligase subunit alpha |
| K01879 | glyS | 2891.6103 | 1.5374 | 1.01E-22 | 1.00E-21 | glycine--tRNA ligase subunit beta |
| K01881 | PARS, proS | 4645.3770 | -1.7461 | 6.23E-19 | 5.07E-18 | proline--tRNA ligase |
| K01892 | HARS, hisS | 3613.0415 | -1.3084 | 2.07E-06 | 6.09E-06 | histidine--tRNA ligase |
| K01915 | glnA, GLUL | 10158.0122 | 1.5620 | 8.85E-06 | 2.42E-05 | type I glutamate--ammonia ligase |
| K01916 | nadE | 1921.9937 | 1.4306 | 7.38E-25 | 8.34E-24 | ammonia-dependent NAD(+) synthetase |
| K01919 | gshA | 381.2654 | -1.3283 | 6.92E-12 | 3.26E-11 | gamma-glutamylcysteine synthetase |
| K01923 | purC | 919.2687 | -1.3156 | 1.01E-09 | 3.96E-09 | phosphoribosylaminoimidazolesuccinocarboxamide synthase |
| K01924 | murC | 5592.1393 | -1.1723 | 5.54E-05 | 0.000135 | UDP-N-acetylmuramate--L-alanine ligase |
| K01933 | purM | 787.1927 | 2.2498 | 2.64E-29 | 4.04E-28 | phosphoribosylformylglycinamidine cyclo-ligase |
| K01945 | purD | 1566.7360 | 1.2658 | 2.26E-11 | 1.02E-10 | phosphoribosylamine--glycine ligase |
| K01952 | purL, PFAS | 2374.8659 | 1.4555 | 2.43E-09 | 9.30E-09 | phosphoribosylformylglycinamidine synthase |
| K01955 | carB, CPA2 | 5386.7604 | 3.2671 | 1.08E-87 | 1.39E-85 | carbamoyl-phosphate synthase large subunit |
| K01955 | carB, CPA2 | 4150.0875 | 1.0444 | 5.01E-11 | 2.19E-10 | ATP-grasp domain-containing protein |
| K01956 | carA, CPA1 | 2687.7294 | 3.3297 | 4.07E-151 | 1.84E-148 | glutamine-hydrolyzing carbamoyl-phosphate synthase small subunit |
| K01999 | livK | 3160.1690 | -2.1714 | 1.59E-31 | 2.76E-30 | ABC transporter substrate-binding protein |
| K02000 | proV | 1894.4183 | -2.3828 | 1.01E-40 | 2.74E-39 | glycine betaine/L-proline ABC transporter ATP-binding protein |
| K02001 | proW | 1707.9692 | -2.1608 | 1.83E-37 | 4.30E-36 | ABC transporter permease/substrate binding protein |
| K02078 | acpP | 140.0108 | -1.3113 | 3.09E-06 | 8.83E-06 | acyl carrier protein |
| K02108 | ATPF0A, atpB | 1297.0538 | -1.0702 | 0.00106 | 0.002133 | F0F1 ATP synthase subunit A |
| K02111 | ATPF1A, atpA | 6694.5320 | -1.2388 | 1.82E-13 | 9.85E-13 | F0F1 ATP synthase subunit alpha |
| K02113 | ATPF1D, atpH | 927.5657 | -1.1238 | 0.001187 | 0.002365 | F0F1 ATP synthase subunit delta |
| K02114 | ATPF1E, atpC | 1296.9656 | -1.3941 | 1.44E-12 | 7.08E-12 | F0F1 ATP synthase subunit epsilon |
| K02171 | blaI | 4470.1038 | 5.0571 | 2.14E-43 | 6.66E-42 | CopY/TcrY family copper transport repressor |
| K02171 | blaI | 7360.5732 | -2.2567 | 2.58E-18 | 2.03E-17 | SMEK domain-containing protein |
| K02314 | dnaB | 1827.8095 | 1.1069 | 2.98E-12 | 1.44E-11 | replicative DNA helicase |
| K02337 | dnaE | 7284.3478 | 1.6497 | 3.14E-55 | 1.77E-53 | DNA polymerase III subunit alpha |
| K02340 | holA | 512.7511 | -2.2983 | 1.22E-12 | 6.00E-12 | DNA polymerase III subunit delta |
| K02341 | holB | 952.3585 | -1.6865 | 3.42E-12 | 1.64E-11 | DNA polymerase III subunit delta' |
| K02343 | dnaX | 92418.6314 | 5.2538 | 5.61E-118 | 1.01E-115 | DNA polymerase III subunit gamma/tau |
| K02371 | fabK | 2009.0101 | -1.7343 | 1.10E-36 | 2.36E-35 | mutanobactin A biosynthesis reductase MubJ |
| K02371 | fabK | 4248.0257 | -1.1118 | 6.31E-16 | 4.29E-15 | enoyl-[acyl-carrier-protein] reductase FabK |
| K02435 | gatC, GATC | 141.6719 | -1.1260 | 1.18E-06 | 3.57E-06 | Asp-tRNA(Asn)/Glu-tRNA(Gln) amidotransferase subunit GatC |
| K02500 | hisF | 340.2201 | 2.1877 | 5.00E-17 | 3.65E-16 | imidazole glycerol phosphate synthase subunit HisF |
| K02501 | hisH | 100.7613 | 2.3056 | 2.54E-12 | 1.23E-11 | imidazole glycerol phosphate synthase subunit HisH |
| K02502 | hisZ | 137.0265 | 1.7663 | 5.95E-10 | 2.40E-09 | ATP phosphoribosyltransferase regulatory subunit |
| K02744 | PTS-Aga-EIIA, agaF | 321.7240 | -1.0586 | 8.54E-05 | 0.000204 | PTS fructose transporter subunit IIA |
| K02757 | PTS-Bgl-EIIC, bglF, bglP | 169.4105 | 1.9892 | 5.43E-08 | 1.86E-07 | PTS transporter subunit EIIC |
| K02761 | PTS-Cel-EIIC, celB, chbC | 505.7134 | 1.9199 | 9.19E-13 | 4.69E-12 | PTS sugar transporter subunit IIC |
| K02768 | PTS-Fru-EIIA, fruB | 23.9146 | 1.7931 | 0.000718 | 0.001485 | PTS transporter subunit EIIA |
| K02770 | PTS-Fru-EIIC, fruA | 2858.4674 | 1.2372 | 6.34E-16 | 4.29E-15 | PTS transporter subunit EIIA |
| K02770 | PTS-Fru-EIIC, fruA | 130.5058 | 2.1314 | 1.63E-06 | 4.82E-06 | PTS transporter subunit EIIC |
| K02786 | PTS-Lac-EIIA, lacF | 23.7676 | 2.3237 | 2.90E-05 | 7.35E-05 | PTS lactose/cellobiose transporter subunit IIA |
| K02788 | PTS-Lac-EIIC, lacE | 362.0821 | 2.1978 | 1.60E-08 | 5.69E-08 | PTS transporter subunit EIIC |
| K02794 | PTS-Man-EIIB, manX | 1570.6092 | -1.4444 | 3.33E-14 | 1.91E-13 | PTS sugar transporter subunit IIB |
| K02794 | PTS-Man-EIIB, manX | 331.0178 | 1.7710 | 3.40E-12 | 1.63E-11 | PTS mannose transporter subunit IIAB |
| K02794 | PTS-Man-EIIB, manX | 357.3431 | 1.2375 | 0.002102 | 0.004019 | PTS sugar transporter subunit IIB |
| K02795 | PTS-Man-EIIC, manY | 759.0729 | 2.1070 | 2.18E-14 | 1.28E-13 | PTS mannose/fructose/sorbose transporter subunit IIC |
| K02796 | PTS-Man-EIID, manZ | 2314.5860 | 1.7248 | 2.03E-09 | 7.80E-09 | PTS mannose/fructose/sorbose transporter family subunit IID |
| K02798 | PTS-Mtl-EIIA, mtlA, cmtB | 111.1071 | -1.1621 | 2.34E-06 | 6.77E-06 | PTS sugar transporter subunit IIA |
| K02819 | PTS-Tre-EIIC, treB | 96.8888 | 2.1135 | 2.54E-16 | 1.77E-15 | PTS system trehalose-specific EIIBC component |
| K02822 | PTS-Ula-EIIB, ulaB, sgaB | 513.5648 | -1.1783 | 2.49E-09 | 9.48E-09 | PTS sugar transporter subunit IIB |
| K02823 | pyrDII | 242.8316 | 2.8354 | 4.48E-31 | 7.72E-30 | dihydroorotate dehydrogenase electron transfer subunit |
| K02825 | pyrR | 364.3462 | 3.4525 | 5.98E-46 | 2.12E-44 | bifunctional pyr operon transcriptional regulator/uracil phosphoribosyltransferase PyrR |
| K02863 | RP-L1, MRPL1, rplA | 2451.4325 | -2.5314 | 1.02E-51 | 4.87E-50 | 50S ribosomal protein L1 |
| K02867 | RP-L11, MRPL11, rplK | 771.2797 | -1.7687 | 2.26E-06 | 6.57E-06 | 50S ribosomal protein L11 |
| K02879 | RP-L17, MRPL17, rplQ | 68.1865 | -1.1689 | 0.014837 | 0.024306 | 50S ribosomal protein L17 |
| K02884 | RP-L19, MRPL19, rplS | 1350.3831 | -1.3473 | 1.11E-23 | 1.17E-22 | 50S ribosomal protein L19 |
| K02909 | RP-L31, rpmE | 1004.1367 | -2.3054 | 1.85E-35 | 3.71E-34 | type B 50S ribosomal protein L31 |
| K02911 | RP-L32, MRPL32, rpmF | 129.9458 | -3.3996 | 5.41E-25 | 6.19E-24 | 50S ribosomal protein L32 |
| K02913 | RP-L33, MRPL33, rpmG | 13.9954 | -2.7502 | 2.63E-06 | 7.56E-06 | 50S ribosomal protein L33 |
| K02913 | RP-L33, MRPL33, rpmG | 11.3161 | -1.5750 | 0.001763 | 0.003414 | 50S ribosomal protein L33 |
| K02935 | RP-L7, MRPL12, rplL | 216.6188 | -1.2259 | 6.50E-08 | 2.20E-07 | 50S ribosomal protein L7/L12 |
| K02939 | RP-L9, MRPL9, rplI | 491.5275 | 1.8334 | 2.22E-27 | 2.86E-26 | 50S ribosomal protein L9 |
| K02956 | RP-S15, MRPS15, rpsO | 791.5855 | -2.3860 | 5.62E-59 | 3.39E-57 | 30S ribosomal protein S15 |
| K02963 | RP-S18, MRPS18, rpsR | 589.3684 | -1.6285 | 1.03E-32 | 1.88E-31 | 30S ribosomal protein S18 |
| K02967 | RP-S2, MRPS2, rpsB | 6917.8439 | -1.6717 | 4.88E-07 | 1.53E-06 | 30S ribosomal protein S2 |
| K02968 | RP-S20, rpsT | 274.7148 | -1.1854 | 4.58E-11 | 2.03E-10 | 30S ribosomal protein S20 |
| K02970 | RP-S21, MRPS21, rpsU | 362.0294 | 1.1521 | 6.50E-10 | 2.61E-09 | 30S ribosomal protein S21 |
| K02986 | RP-S4, rpsD | 1307.5931 | -1.0606 | 1.01E-14 | 6.07E-14 | 30S ribosomal protein S4 |
| K02996 | RP-S9, MRPS9, rpsI | 775.9767 | -1.3540 | 7.70E-19 | 6.21E-18 | 30S ribosomal protein S9 |
| K03040 | rpoA | 985.4186 | -1.1255 | 0.000711 | 0.001472 | DNA-directed RNA polymerase subunit alpha |
| K03048 | rpoE | 7756.2173 | -2.5779 | 3.01E-136 | 9.07E-134 | DNA-directed RNA polymerase subunit delta |
| K03073 | secE | 8.0156 | -1.2455 | 0.02157 | 0.03422 | preprotein translocase subunit SecE |
| K03100 | lepB | 211.2896 | -1.8291 | 7.32E-21 | 6.55E-20 | signal peptidase I |
| K03111 | ssb | 738.9866 | -1.4576 | 2.79E-15 | 1.78E-14 | single-stranded DNA-binding protein |
| K03210 | yajC | 1258.5996 | -2.0496 | 1.78E-19 | 1.51E-18 | preprotein translocase subunit YajC |
| K03367 | dltA | 6302.6158 | 1.2458 | 1.27E-10 | 5.39E-10 | D-alanine--poly(phosphoribitol) ligase subunit DltA |
| K03431 | glmM | 6721.9462 | 1.4637 | 1.36E-09 | 5.30E-09 | phosphoglucosamine mutase |
| K03471 | rnhC | 854.4189 | -1.4043 | 2.26E-05 | 5.79E-05 | ribonuclease HIII |
| K03475 | PTS-Ula-EIIC, ulaA, sgaT | 221.4739 | 1.3876 | 1.36E-05 | 3.60E-05 | PTS ascorbate transporter subunit IIC |
| K03523 | bioY | 14.1964 | 1.4058 | 0.011267 | 0.018917 | biotin transporter BioY |
| K03531 | ftsZ | 11725.0178 | 1.1399 | 2.80E-07 | 9.05E-07 | cell division protein FtsZ |
| K03553 | recA | 10409.8927 | -1.7378 | 1.31E-28 | 1.83E-27 | recombinase RecA |
| K03555 | mutS | 2086.8163 | 1.6509 | 4.55E-41 | 1.25E-39 | DNA mismatch repair protein MutS |
| K03575 | mutY | 986.6195 | -1.5199 | 1.25E-17 | 9.40E-17 | A/G-specific adenine glycosylase |
| K03588 | ftsW, spoVE | 2633.4835 | -1.2608 | 0.000195 | 0.000438 | FtsW/RodA/SpoVE family cell cycle protein |
| K03589 | ftsQ | 8912.6553 | -1.3262 | 2.91E-44 | 9.40E-43 | FtsQ-type POTRA domain-containing protein |
| K03596 | lepA | 775.4579 | 1.1504 | 1.10E-06 | 3.32E-06 | elongation factor 4 |
| K03639 | moaA, CNX2 | 232.4529 | -1.2865 | 3.44E-15 | 2.17E-14 | YccF domain-containing protein |
| K03648 | UNG, UDG | 288.6731 | 1.4048 | 2.25E-10 | 9.34E-10 | uracil-DNA glycosylase |
| K03671 | trxA | 410.0155 | -1.4729 | 4.11E-18 | 3.18E-17 | thioredoxin |
| K03671 | trxA | 345.0748 | -1.5272 | 6.55E-13 | 3.36E-12 | conjugal transfer protein TraF |
| K03695 | clpB | 899.5309 | 2.0563 | 3.50E-24 | 3.81E-23 | AAA domain-containing protein |
| K03702 | uvrB | 5312.8120 | 1.4249 | 2.12E-05 | 5.46E-05 | excinuclease ABC subunit B |
| K03739 | dltB | 5485.4891 | 1.3847 | 8.94E-12 | 4.16E-11 | D-alanyl-lipoteichoic acid biosynthesis protein DltB |
| K03740 | dltD | 2437.9288 | 1.2684 | 3.20E-13 | 1.70E-12 | D-alanyl-lipoteichoic acid biosynthesis protein DltD |
| K03767 | PPIA | 300.3579 | -1.3907 | 6.06E-10 | 2.43E-09 | peptidyl-prolyl cis-trans isomerase |
| K03783 | punA, PNP | 1395.0439 | 1.1100 | 3.37E-08 | 1.17E-07 | purine-nucleoside phosphorylase |
| K04043 | dnaK, HSPA9 | 208752.1345 | 1.7708 | 1.96E-05 | 5.05E-05 | molecular chaperone DnaK |
| K04072 | adhE | 10842.2153 | 3.0851 | 1.57E-11 | 7.16E-11 | bifunctional acetaldehyde-CoA/alcohol dehydrogenase |
| K04077 | groEL, HSPD1 | 260865.3746 | 1.1810 | 0.004135 | 0.007495 | chaperonin GroEL |
| K04567 | KARS, lysS | 300.9702 | -1.5155 | 1.02E-12 | 5.14E-12 | RNA-binding S4 domain-containing protein |
| K05339 | lrgB | 336.5814 | 3.0807 | 4.40E-42 | 1.26E-40 | LrgB family protein |
| K05363 | murM | 2601.7640 | -1.0380 | 1.27E-11 | 5.84E-11 | aminoacyltransferase |
| K05823 | dapL | 903.0072 | 1.5003 | 6.14E-25 | 6.98E-24 | N-acetyldiaminopimelate deacetylase |
| K05845 | opuC | 1297.7297 | 1.7530 | 7.87E-43 | 2.37E-41 | osmoprotectant ABC transporter substrate-binding protein |
| K05845 | opuC | 3544.1527 | 2.0586 | 7.51E-16 | 5.05E-15 | ABC transporter permease/substrate-binding protein |
| K05846 | opuBD | 324.6281 | 1.5165 | 1.79E-13 | 9.74E-13 | ABC transporter permease |
| K05846 | opuBD | 525.4787 | 1.8834 | 6.66E-09 | 2.45E-08 | ABC transporter permease |
| K05847 | opuA | 1341.0071 | 2.3359 | 5.18E-21 | 4.73E-20 | ABC transporter ATP-binding protein |
| K05964 | citX | 111.1520 | 1.7297 | 3.24E-13 | 1.72E-12 | citrate lyase holo-[acyl-carrier protein] synthase |
| K06131 | clsA_B | 1462.0541 | 1.1579 | 0.000833 | 0.001703 | cardiolipin synthase |
| K06133 | LYS5, acpT | 928.0087 | -1.9003 | 1.56E-26 | 1.93E-25 | mutanobactin A biosynthesis phosphopantetheinyl transferase MubP |
| K06223 | dam | 2422.4713 | -2.4649 | 1.37E-56 | 8.00E-55 | DNA adenine methylase |
| K06229 | SUFU | 1242.6668 | 2.3771 | 1.47E-38 | 3.73E-37 | suppressor of fused domain protein |
| K06881 | nrnA | 1122.5584 | -2.3560 | 1.18E-32 | 2.14E-31 | bifunctional oligoribonuclease/PAP phosphatase NrnA |
| K07141 | mocA | 26.0242 | -1.4167 | 0.000599 | 0.001255 | hypothetical protein |
| K07213 | ATOX1, ATX1, copZ, golB | 125.7875 | 4.4629 | 1.90E-35 | 3.78E-34 | copper chaperone CopZ |
| K07260 | vanY | 31827.5713 | 2.9301 | 1.81E-07 | 5.93E-07 | YSIRK-type signal peptide-containing protein |
| K07407 | E3.2.1.22B, galA, rafA | 1261.7104 | 1.6189 | 6.41E-35 | 1.25E-33 | alpha-galactosidase |
| K07462 | recJ | 1219.8773 | -1.1975 | 7.71E-07 | 2.37E-06 | single-stranded-DNA-specific exonuclease RecJ |
| K07652 | vicK | 1841.2528 | -1.5959 | 1.47E-25 | 1.74E-24 | cell wall metabolism sensor histidine kinase VicK |
| K07668 | vicR | 1380.8713 | -2.0317 | 5.04E-34 | 9.69E-33 | response regulator transcription factor |
| K07704 | lytS | 826.8219 | 1.0751 | 6.15E-07 | 1.92E-06 | sensor protein LytS |
| K07755 | - | 5990.7467 | -4.0959 | 6.89E-153 | 4.15E-150 | class I SAM-dependent methyltransferase |
| K08483 | PTS-EI.PTSI, ptsI | 26301.3222 | 1.2941 | 7.85E-08 | 2.63E-07 | phosphoenolpyruvate--protein phosphotransferase |
| K08680 | menH | 604.1398 | -2.1695 | 6.60E-21 | 5.96E-20 | mutanobactin A biosynthesis alpha/beta hydrolase MubM |
| K08968 | msrC | 6998.2667 | 5.4710 | 1.29E-124 | 2.59E-122 | GAF domain-containing protein |
| K09457 | queF | 16.2887 | -1.3974 | 0.003715 | 0.006809 | NADPH-dependent 7-cyano-7-deazaguanine reductase QueF |
| K09698 | gltX | 2240.2842 | -1.1274 | 8.50E-07 | 2.60E-06 | glutamate--tRNA ligase |
| K09811 | ftsX | 3997.7913 | 1.4265 | 2.44E-35 | 4.80E-34 | ABC transporter permease |
| K09812 | ftsE | 5554.7327 | 1.1268 | 6.26E-22 | 5.89E-21 | cell division ATP-binding protein FtsE |
| K09816 | znuB | 315.5609 | 1.3930 | 4.73E-11 | 2.08E-10 | metal ABC transporter permease |
| K09817 | znuC | 1688.4578 | 1.7954 | 2.75E-25 | 3.21E-24 | metal ABC transporter ATP-binding protein |
| K10009 | ABC.CYST.P | 59.0688 | 1.8188 | 9.31E-09 | 3.35E-08 | amino acid ABC transporter permease |
| K10039 | ABC.GLN1.S | 3303.5877 | 2.4677 | 3.02E-24 | 3.31E-23 | transporter substrate-binding domain-containing protein |
| K10040 | ABC.GLN1.P | 1185.1506 | 3.1603 | 3.86E-22 | 3.71E-21 | amino acid ABC transporter permease |
| K10040 | ABC.GLN1.P | 1266.4121 | 2.9367 | 1.42E-20 | 1.23E-19 | amino acid ABC transporter permease |
| K10041 | ABC.GLN1.A | 3166.7204 | 1.7246 | 4.12E-11 | 1.84E-10 | amino acid ABC transporter ATP-binding protein |
| K10112 | msmX, msmK, malK, sugC, ggtA, msiK | 428.1602 | 2.1084 | 9.80E-29 | 1.41E-27 | sn-glycerol-3-phosphate ABC transporter ATP-binding protein UgpC |
| K10112 | msmX, msmK, malK, sugC, ggtA, msiK | 110.6090 | 1.3295 | 3.87E-06 | 1.10E-05 | sn-glycerol-3-phosphate ABC transporter ATP-binding protein UgpC |
| K10117 | msmE | 1067.7712 | 1.8400 | 1.43E-28 | 1.97E-27 | extracellular solute-binding protein |
| K10118 | msmF | 536.0791 | 1.6304 | 1.31E-26 | 1.64E-25 | sugar ABC transporter permease |
| K10119 | msmG | 1325.0026 | 2.3755 | 4.07E-24 | 4.41E-23 | carbohydrate ABC transporter permease |
| K10563 | mutM, fpg | 1067.3378 | 1.3100 | 1.41E-09 | 5.47E-09 | DNA-formamidopyrimidine glycosylase |
| K10679 | nfnB, nfsB | 3116.6607 | 1.8071 | 3.39E-10 | 1.39E-09 | NAD(P)H-dependent oxidoreductase |
| K11041 | eta | 40.4999 | 2.1479 | 4.76E-11 | 2.09E-10 | TDT family transporter |
| K11050 | ABC-2.CYL.A, cylA | 702.3108 | 2.4841 | 5.51E-15 | 3.39E-14 | ABC transporter ATP-binding protein |
| K11051 | ABC-2.CYL.P, cylB | 365.1691 | 2.4225 | 2.31E-09 | 8.85E-09 | ABC transporter permease |
| K11175 | purN | 193.8483 | 2.1522 | 1.13E-12 | 5.64E-12 | phosphoribosylglycinamide formyltransferase |
| K11521 | K11521, manR | 679.8467 | 1.0403 | 9.28E-05 | 0.000219 | response regulator transcription factor |
| K11621 | liaG | 264.9101 | 1.5755 | 0.000894 | 0.001815 | DUF1700 domain-containing protein |
| K11622 | liaF | 85.0930 | 1.1614 | 0.001558 | 0.003054 | transporter |
| K11749 | rseP | 1658.3725 | -1.4601 | 1.02E-07 | 3.37E-07 | RIP metalloprotease |
| K11754 | folC | 670.1777 | -3.2471 | 5.85E-29 | 8.67E-28 | bifunctional folylpolyglutamate synthase/dihydrofolate synthase |
| K11754 | folC | 3252.7958 | 1.7676 | 2.34E-08 | 8.26E-08 | bifunctional folylpolyglutamate synthase/dihydrofolate synthase |
| K12296 | comX1_2 | 48.1075 | -1.6642 | 1.30E-07 | 4.27E-07 | sigma-70 family RNA polymerase sigma factor |
| K12554 | murN | 1502.3553 | -1.0795 | 2.29E-10 | 9.49E-10 | aminoacyltransferase |
| K12574 | rnj | 2727.9355 | 1.4177 | 2.08E-27 | 2.71E-26 | ribonuclease J |
| K13677 | dgs, bgsA | 4802.6477 | 1.3798 | 3.84E-06 | 1.09E-05 | glycosyltransferase |
| K13732 | fnbA | 11047.8225 | 1.2639 | 0.000252 | 0.000553 | glucan-binding protein |
| K14155 | patB, malY | 619.5922 | -1.7174 | 1.54E-15 | 1.00E-14 | pyridoxal phosphate-dependent aminotransferase |
| K14155 | patB, malY | 46.7945 | 1.0399 | 0.001051 | 0.002117 | GNAT family N-acetyltransferase |
| K14188 | dltC | 430.6937 | 1.5740 | 1.81E-16 | 1.28E-15 | D-alanine--poly(phosphoribitol) ligase subunit DltC |
| K14194 | sdrC_D_E | 27977.0460 | 1.9722 | 1.07E-10 | 4.54E-10 | LPXTG cell wall anchor domain-containing protein |
| K14698 | irtA | 254.4054 | 2.4142 | 9.16E-21 | 8.07E-20 | ABC transporter ATP-binding protein |
| K14699 | irtB | 182.5964 | 2.1702 | 1.05E-24 | 1.17E-23 | ABC transporter ATP-binding protein |
| K14982 | ciaH | 5358.1135 | 1.7635 | 2.68E-14 | 1.56E-13 | HAMP domain-containing histidine kinase |
| K14982 | ciaH | 30734.6041 | 1.0216 | 3.49E-13 | 1.84E-12 | HAMP domain-containing histidine kinase |
| K14983 | ciaR | 13068.5123 | 1.5381 | 1.41E-28 | 1.96E-27 | response regulator transcription factor |
| K15634 | gpmB | 395.6594 | 1.1389 | 1.29E-05 | 3.42E-05 | histidine phosphatase family protein |
| K15661 | ituA, mycA, bmyA | 1506.1114 | -2.0319 | 1.83E-11 | 8.35E-11 | mutanobactin A non-ribosomal peptide synthetase MubE |
| K15770 | cycB, ganO | 908.6682 | 1.0732 | 0.000696 | 0.001445 | extracellular solute-binding protein |
| K15771 | ganP | 161.9440 | 1.9484 | 8.15E-25 | 9.15E-24 | sugar ABC transporter permease |
| K15772 | ganQ | 38.5120 | 1.7568 | 3.00E-06 | 8.59E-06 | sugar ABC transporter permease |
| K15860 | ciaX | 33.0660 | 1.6219 | 0.000181 | 0.00041 | hypothetical protein |
| K15986 | ppaC | 2620.1972 | 1.4586 | 7.41E-21 | 6.59E-20 | CBS domain-containing protein |
| K16093 | bacA | 5176.7191 | -3.3135 | 4.91E-47 | 1.85E-45 | non-ribosomal peptide synthetase |
| K16093 | bacA | 5590.5362 | -2.8973 | 4.57E-26 | 5.54E-25 | non-ribosomal peptide synthetase |
| K16123 | tycB | 3856.5909 | -2.9332 | 8.50E-31 | 1.45E-29 | mutanobactin A non-ribosomal peptide synthetase MubB |
| K16124 | tycC | 4673.6994 | -3.1681 | 1.80E-32 | 3.23E-31 | non-ribosomal peptide synthetase |
| K16124 | tycC | 1354.5624 | -2.5868 | 5.89E-19 | 4.84E-18 | mutanobactin A polyketide synthase MubH |
| K16957 | tcyK | 269.8595 | -2.8339 | 1.35E-10 | 5.68E-10 | amino acid ABC transporter substrate-binding protein |
| K16958 | tcyL | 214.0915 | -3.0689 | 2.23E-10 | 9.30E-10 | amino acid ABC transporter permease |
| K16959 | tcyM | 141.5006 | -3.3664 | 2.55E-24 | 2.83E-23 | amino acid ABC transporter permease |
| K16960 | tcyN | 106.0867 | -3.3716 | 3.39E-16 | 2.34E-15 | amino acid ABC transporter ATP-binding protein |
| K17077 | - | 416.6887 | 1.4479 | 4.14E-09 | 1.55E-08 | amino acid ABC transporter permease |
| K17363 | urdA | 698.7050 | 2.7171 | 7.50E-10 | 2.99E-09 | NAD(P)H-dependent oxidoreductase |
| K17363 | urdA | 11216.5054 | 2.2461 | 4.39E-08 | 1.51E-07 | flavocytochrome c |
| K17363 | urdA | 53.2034 | 1.2122 | 0.000374 | 0.000806 | FAD-dependent oxidoreductase |
| K17363 | urdA | 3726.1297 | 1.0605 | 0.000619 | 0.001293 | NAD(P)H-dependent oxidoreductase |
| K17467 | PTS-Dga-EIID, dgaD | 5900.0267 | -1.1892 | 8.08E-05 | 0.000194 | PTS system mannose/fructose/sorbose family transporter subunit IID |
| K17686 | copA, ATP7 | 43822.6553 | 5.4018 | 9.66E-64 | 7.27E-62 | copper-translocating P-type ATPase |
| K17828 | pyrDI | 79.2003 | 2.3453 | 5.58E-19 | 4.60E-18 | dihydroorotate dehydrogenase |
| K18136 | acrR | 240.8578 | 2.5206 | 8.99E-19 | 7.22E-18 | TetR/AcrR family transcriptional regulator |
| K18474 | fabM | 2886.6328 | -1.9900 | 3.94E-77 | 4.75E-75 | enoyl-CoA hydratase |
| K18682 | rny | 11545.5498 | -2.7910 | 3.25E-74 | 3.67E-72 | ribonuclease Y |
| K18887 | efrA | 652.2300 | 2.0467 | 5.38E-15 | 3.32E-14 | ABC transporter ATP-binding protein |
| K18888 | efrB | 531.9973 | 1.8986 | 5.02E-19 | 4.18E-18 | ABC transporter ATP-binding protein |
| K18889 | mdlA, smdA | 258.1414 | 2.4023 | 5.99E-18 | 4.60E-17 | ATP-binding cassette domain-containing protein |
| K18890 | mdlB, smdB | 384.6885 | 2.1990 | 1.01E-14 | 6.07E-14 | ABC transporter ATP-binding protein |
| K18941 | arlR | 1585.3064 | -2.5462 | 3.23E-42 | 9.41E-41 | response regulator transcription factor |
| K19003 | mgdA | 255.6484 | -1.1760 | 2.22E-07 | 7.21E-07 | glycosyltransferase family 2 protein |
| K19083 | braD, bceA | 162.8377 | 1.5487 | 4.33E-13 | 2.28E-12 | ABC transporter ATP-binding protein |
| K19084 | braE, bceB | 565.1253 | 2.4232 | 4.08E-65 | 3.51E-63 | ABC transporter permease |
| K19710 | E2.7.7.53 | 195.6341 | -1.1077 | 7.63E-07 | 2.36E-06 | HIT family protein |
| K20108 | PTS-Mal-EIIC, malT | 1989.1116 | 1.9248 | 8.08E-12 | 3.78E-11 | PTS transporter subunit EIIC |
| K20265 | gadC | 17.2204 | -1.2250 | 0.007833 | 0.013493 | APC family permease |
| K20341 | blpL, cipI | 1077.1717 | -1.9885 | 7.20E-26 | 8.62E-25 | hypothetical protein |
| K20342 | comR | 4891.5160 | -1.6750 | 8.11E-12 | 3.79E-11 | helix-turn-helix transcriptional regulator |
| K20342 | comR | 3270.5833 | -1.3868 | 1.31E-06 | 3.96E-06 | helix-turn-helix transcriptional regulator |
| K20373 | rgg2 | 2423.4682 | -2.5376 | 6.01E-30 | 9.69E-29 | Rgg/GadR/MutR family transcriptional regulator |
| K20374 | rgg3 | 1613.3877 | -2.5834 | 7.36E-60 | 4.75E-58 | Rgg/GadR/MutR family transcriptional regulator |
| K20492 | nisG, spaG, cprC | 42.0453 | 1.3102 | 0.000233 | 0.000514 | lantibiotic immunity ABC transporter MutG family permease subunit |
| K21064 | ycsE, yitU, ywtE | 6231.1634 | 2.6199 | 2.04E-26 | 2.51E-25 | Cof-type HAD-IIB family hydrolase |
| K21064 | ycsE, yitU, ywtE | 1403.4213 | -1.1301 | 1.04E-12 | 5.24E-12 | sugar-phosphatase |
| K21064 | ycsE, yitU, ywtE | 483.0600 | -1.5043 | 1.76E-05 | 4.59E-05 | Cof-type HAD-IIB family hydrolase |
| K21977 | coaB | 318.8928 | -3.0977 | 6.86E-24 | 7.29E-23 | phosphopantothenate--cysteine ligase |
| K22212 | mleA, mleS | 2233.2620 | 3.4974 | 2.53E-25 | 2.97E-24 | NAD-dependent malic enzyme |
| K22223 | pgp | 179.7200 | 1.4874 | 8.21E-08 | 2.74E-07 | Cof-type HAD-IIB family hydrolase |
| K22227 | ahbD | 669.7119 | -1.8584 | 4.63E-08 | 1.59E-07 | radical SAM protein |
| K22305 | psp | 282.4845 | -1.4298 | 2.07E-18 | 1.64E-17 | histidine phosphatase family protein |

**
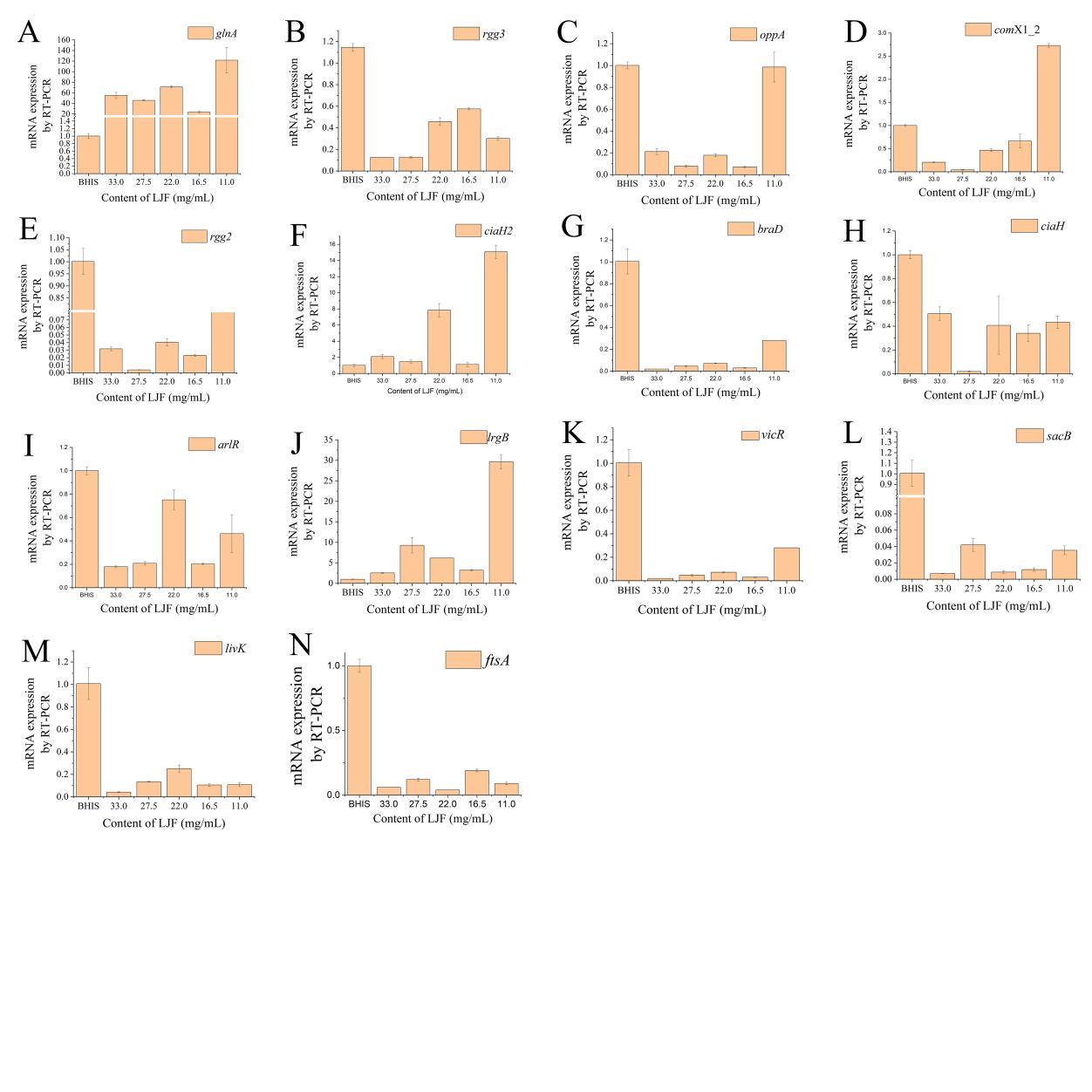
**

**Figure S3.** The mRNA expression level of DEGs
